# PDGFRα^+^ stromal adipocyte progenitors transition into epithelial cells during lobulo-alveologenesis in the murine mammary gland

**DOI:** 10.1038/s41467-019-09748-z

**Published:** 2019-04-15

**Authors:** Purna A. Joshi, Paul D. Waterhouse, Katayoon Kasaian, Hui Fang, Olga Gulyaeva, Hei Sook Sul, Paul C. Boutros, Rama Khokha

**Affiliations:** 1 Princess Margaret Cancer Centre, Toronto, ON M5G 1L7 Canada; 2 Ontario Institute for Cancer Research, Toronto, ON M5G 0A3 Canada; 3 Endocrinology Program, University of California, Berkeley, CA 94720 USA; 4 Department of Nutritional Science & Toxicology, University of California, Berkeley, CA 94720 USA; 5 Department of Medical Biophysics, University of Toronto, Toronto, ON M5G 1L7 Canada

## Abstract

The mammary gland experiences substantial remodeling and regeneration during development and reproductive life, facilitated by stem cells and progenitors that act in concert with physiological stimuli. While studies have focused on deciphering regenerative cells within the parenchymal epithelium, cell lineages in the stroma that may directly contribute to epithelial biology is unknown. Here we identify, in mouse, the transition of a PDGFRα^+^ mesenchymal cell population into mammary epithelial progenitors. In addition to being adipocyte progenitors, PDGFRα^+^ cells make a de novo contribution to luminal and basal epithelia during mammary morphogenesis. In the adult, this mesenchymal lineage primarily generates luminal progenitors within lobuloalveoli during sex hormone exposure or pregnancy. We identify cell migration as a key molecular event that is activated in mesenchymal progenitors in response to epithelium-derived chemoattractant. These findings demonstrate a stromal reservoir of epithelial progenitors and provide insight into cell origins and plasticity during mammary tissue growth.

## Abstract (web summary)

The origin and source of mammary gland progenitors and how they interact with the adipose‐rich stroma is unclear. Here, the authors identify PDGFRα^+^ adipocyte progenitors in the murine mammary stroma as a mesenchymal cell lineage recruited into the expanding epithelium during development, hormone exposure and pregnancy.

## Introduction

The mammary parenchyma comprises an inner layer of luminal epithelial cells and an outer basal epithelial lineage^1^. The luminal lineage can differentiate into lobuloalveolar structures during the female reproductive cycle and become milk-secreting sacs following pregnancy. The basal lineage gives rise to differentiated myoepithelial cells that are contractile and aid in milk expulsion. Early mammary development as well as adult tissue growth and regeneration rely on stem cells and progenitors to generate epithelial lineages upon physiological demand. Research on mammary epithelial precursors has been fueled by therapeutic challenges in breast cancer arising from breast cancer heterogeneity and evidence suggests that mammary stem cells or their progenitors are putative cells of origin in distinct breast cancer subtypes^2^. Work from several groups^3–12^ has yielded knowledge on the existence, characteristics, potency, location, and regulation of mammary stem and progenitor cells within epithelial lineages.

The mammary epithelium is embedded in an adipose-rich stroma that contains haematopoietic, endothelial cells, the extracellular matrix and mesenchymal cells such as fibroblasts and adipocyte precursors. The importance of stromal-epithelial interactions for mammary gland biology and breast cancer has long been appreciated^13,14^. As early as embryonic development, the mesenchyme is known to induce formation of the mammary epithelial bud^13^. The greater part of mammary growth and branching that takes place during postnatal life is dependent on an intricate interplay between the hypothalamic-pituitary-ovarian hormone axis and cell-cell communications where diverse stromal elements play a crucial role^14^. In breast cancer, carcinoma associated fibroblasts in the tumor microenvironment drive tumor growth and metastasis^15^. A significant stromal influence on early cancer development is also evident in studies where exposure of the stroma alone to carcinogens is sufficient to trigger tumorigenesis within the epithelium^16,17^. While mammary stem cells and progenitors are recognized precursors for epithelial expansion, our understanding of the impact of stromal niche cells on these cell populations is rather limited^18,19^. In particular, stromal lineages that directly contribute to the epithelial precursor pool have not been defined.

Adipocytes are abundant in mammary stroma and tissue-ablation studies in mice have inferred the importance of adipocytes in mammary development^20,21^. In white adipose tissue depots, adipocytes have been shown to arise from resident adipocyte progenitors^22,23^. Lineage tracing studies have established Platelet Derived Growth Factor Receptor alpha (PDGFRα) as a marker of adipocyte progenitors that can generate *de novo* functional adipocytes in vivo^24,25^. PDGFRα is expressed by mesenchymal cell populations and is involved in the development of diverse tissues^26,27^.In skin epithelia, adipocyte precursor cells are involved in driving the regenerative hair cycle^28^. The mammary gland is a skin appendage and similar to the hair follicle, it undergoes significant growth and cyclical remodeling in postnatal life^29^. However, dynamics of adipocyte progenitors during mammary epithelial expansion have been unexplored.

Here, we show that PDGFRα marks mesenchymal adipocyte progenitors that form a distinct stromal layer encasing the parenchymal epithelial lineages of the mouse mammary gland. PDGFRα^+^ progeny are present in mammary epithelial lineages from early embryonic development and throughout morphogenesis in postnatal life. These stromal progenitors are recruited into the mammary epithelium during early development and in the adult gland upon steroid sex hormone exposure or pregnancy. We find that mesenchymal adipocyte precursors marked by Preadipocyte factor 1 (PREF-1) also transition into adult mammary epithelial cells while mature adipocytes do not. Through combined transplantation and lineage tracing experiments, we demonstrate the mesenchymal-to-epithelial switch of stroma-localized cells during mammary epithelial expansion. PDGFRα^+^ stromal cells exhibit a robust migratory molecular profile in response to sex hormones in vivo. We observe increased expression levels of *Pdgfc*, a PDGFRα ligand, in epithelial cells from hormone-stimulated mice, and illustrate chemotactic migration of PDGFRα^+^ stromal cells following PDGFCC administration. Taken together, this study exposes a PDGFRα^+^ and PREF-1^+^ mesenchymal adipogenic cell pool as a source of epithelial descendents in the expanding mammary gland.

## Results

### PDGFRα marks mesenchymal progenitors in the mammary gland

**Fig. 1:**
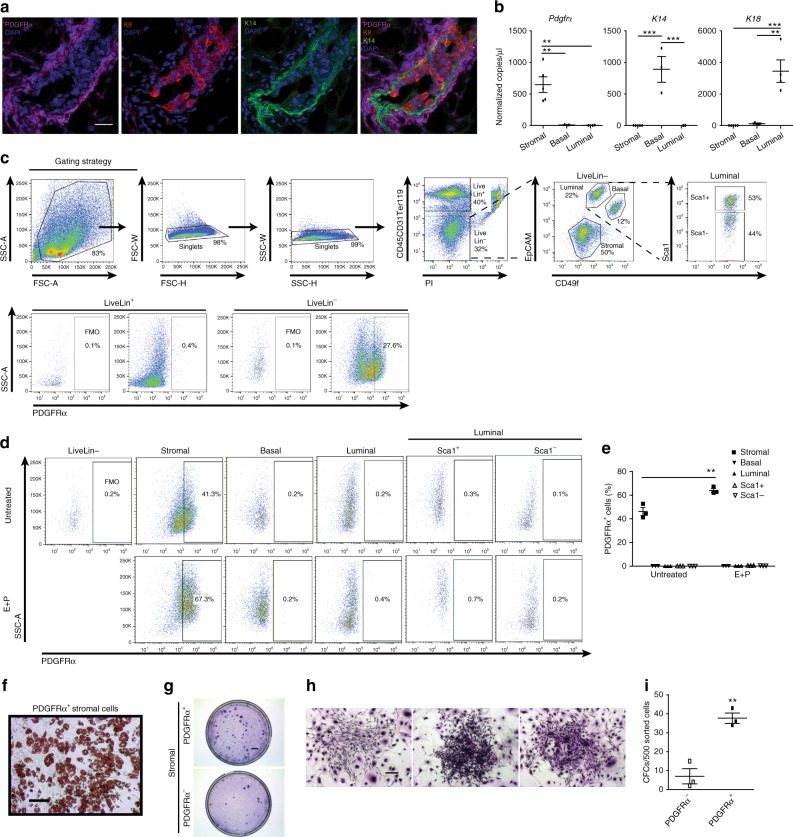
PDGFRα^+^ mammary cells are a stromal adipogenic population enriched in progenitors. **a** Maximum intensity Z-projection of confocal immunofluorescence for luminal (K8; red) and basal (K14; green) epithelial-specific keratins with PDGFRα (magenta) and nuclear DAPI (blue) on a representative adult mouse mammary tissue section (*n* = 3 mice, 5 fields per section); scale bar = 25 µm. **b** Droplet digital PCR analysis of *Pdgfrα* and epithelial keratins *K14*, *K18* normalized to *Gapdh* in fluorescence-activated cell sorting (FACS)-sorted stromal (CD49f^-^EpCAM^-^, *n* = 5) and luminal (CD49f^lo^EpCAM^+^, *n* = 4), basal (CD49f^hi^EpCAM^+^, *n* = 3) mammary epithelial cells. **c** FACS gating strategy used to exclude doublets and dead cells (PI^+^) from analysis of PDGFRα^+^ cells within the lineage^+^ (Lin^+^) population that consists of haematopoietic (CD45^+^), endothelial (CD31^+^) cells and erythrocytes (Ter119^+^), and the Lin^-^ population that consists of mammary stromal and epithelial subpopulations segregated using indicated cell surface markers; fluorescence minus one (FMO) control. **d** Flow cytometry analyses of PDGFRα^+^ cells within distinct mammary subpopulations derived from adult mammary glands of untreated or sex hormone (E+P) treated mice (*n* = 3 mice per group). **e** Quantification of PDGFRα^+^ cells in (d). **f** Image of adipocyte differentiation and lipid accumulation in FACS-sorted PDGFRα^+^ cells as detected by Oil Red O staining (representative of cultured sorted cells from *n*=6 individual mice); scale bar = 100 µm. **g** Colony forming cell (CFC) assay performed on sorted PDGFRα^-^ and PDGFRα^+^ stromal cells (*n* = 6 mice); scale bar = 5 mm. **h** Representative stromal colonies generated by PDGFRα^+^ cells (*n* = 6 mice); scale bar = 200 µm. **i** CFC capacity of PDGFRα^-^ and PDGFRα^+^ cells (*n* = 3 mice). Data represent mean ± s.e.m. *p< 0.05, **p<0.01, ***p<0.001 (*t*-test). Source data are provided as a Source Data file

Given the abundant adipose content in the adult mammary gland, we examined whether PDGFRα can serve as a marker of mammary stromal progenitors. In addition to being scattered throughout the mouse fat pad, PDGFRα^+^ stromal cells formed a concentrated layer closely surrounding the bilayered mammary epithelium, which comprises inner luminal cells and outer basal cells^1^ (Fig. 1a). This distribution persisted during the estrous reproductive cycle or following hormonal stimulation (Supplementary Fig. 1a). Despite the striking proximity of PDGFRα^+^ cells to epithelial structures, co-staining with luminal- and basal-specific cytokeratins showed that PDGFRα does not overlap with epithelial keratins (Fig. 1a). Based on established cell surface marker profiles^3,30^, stromal (CD49f^-^EpCAM^-^), luminal (CD49f^lo^EpCAM^+^) and basal (CD49f^hi^EpCAM^+^) cell fractions were purified using fluorescence-activated cell sorting (FACS) and *Pdgfrα* expression was analyzed by droplet digital PCR (ddPCR) used for detecting low mRNA levels (Fig. 1b). *Pdgfrα* transcripts were only observed in the stromal fraction, and absent in K14-expressing basal and K18-expressing luminal cells. Mice treated with either vehicle or sex hormones (estrogen, E; estrogen and progesterone, E+P) also showed *Pdgfrα* mRNA expression exclusively in stromal cells (Supplementary Fig. 1b). Next, flow cytometry of PDGFRα^+^ cells showed their presence in lineage^-^ cells but not in the lineage^+^ hematopoietic and endothelial populations (Fig. 1c). Within lineage^-^ cells, PDGFRα^+^ cells were significantly present in the stromal mesenchymal subset, but not found in epithelial fractions (basal, luminal, or luminal subpopulations segregated by Sca1 that enriches for estrogen and progesterone receptor positive (ER^+^PR^+^, Sca1^+^) versus negative (ER^-^PR^-^, Sca1^-^) cells and progenitors;^30,31^ Fig. 1d, e). The proportion of PDGFRα^+^ cells in the stroma increased upon hormone stimulation indicative of their hormone sensitivity (Fig. 1e). FACS-sorted PDGFRα^+^ cells differentiated into lipid-laden adipocytes in vitro (Fig. 1f), and were enriched for stromal progenitors as seen through their increased colony forming capacity (Fig. 1g–i). These data identify a PDGFRα^+^ progenitor population that is hormone-sensitive and adipogenic in the mammary stroma.

### Progeny of the Pdgfrα^+^ lineage exists in mammary epithelium

**Fig. 2:**
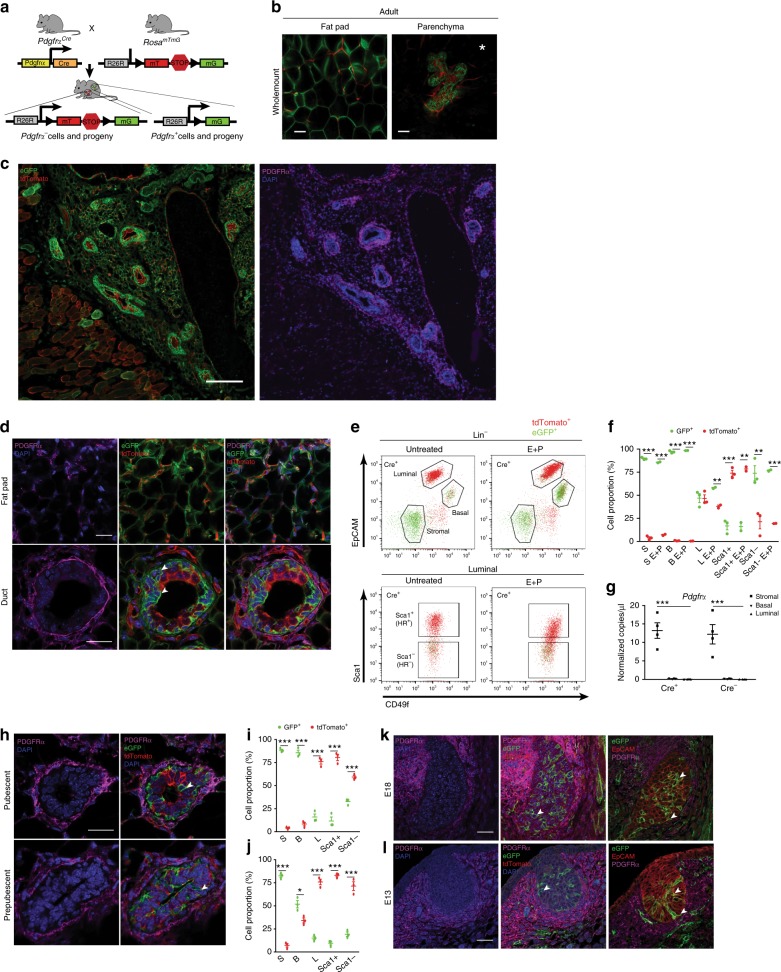
Lineage tracing Pdgfrα^+^ mesenchymal cells reveals GFP-labeled progeny within the mammary epithelium. **a** Lineage tracing model used to determine stromal cell dynamics in the mammary gland. **b** Whole mount confocal images of native GFP (green) and tdTomato (red) fluorescence in the mammary fat pad (left) and epithelia (right; E+P treated) of adult *PdgfrαCre R26mTmG* mice; scale bar = 50 µm. **c** GFP, tdTomato (left) and PDGFRα (magenta), DAPI (blue) (right) immunostaining spanning several epithelial structures in *PdgfrαCre R26mTmG* mammary tissue derived from **b**; scale bar = 200 µm. **d** High magnification image of PDGFRα, GFP and tdTomato in the fat pad and glandular regions from an E+P treated mouse (representative of *n* = 5 adult mice and 5 fields per tissue section); scale bar = 25 µm. **e** FACS profiles of GFP^+^ and tdTomato^+^ cells in mammary subpopulations: stromal, basal, luminal and luminal subsets enriched for hormone receptor (HR) positive (Sca1^+^) and negative (Sca1^-^) cells of adult mammary glands isolated from untreated (*n* = 3) or E+P treated (*n* = 2) mice; **f** Quantification of GFP^+^ and tdTomato^+^ cells in stromal (S), basal (B) and luminal (L) subpopulations shown in **e**. **g** Droplet digital PCR analysis of *Pdgfrα* transcripts normalized to *Gapdh* in FACS-sorted mammary cell fractions from lineage tracing mice (*n* = 4 per group). **h** Immunofluorescent images of *PdgfrαCre R26mTmG* mammary tissue sections from pubescent and prepubescent mice (imaging of *n* = 3 mice per stage and 5 fields per tissue section); scale bar = 25 µm. Quantification of GFP^+^ and tdTomato^+^ cells in mammary subsets of **i** pubescent (*n* = 3) and **j** prepubescent (*n* = 3) mice. PDGFRα^+^ cells (magenta), GFP^+^ progeny (green) and epithelial EpCAM (red) in the embryonic mammary gland at embryonic day 18 **k** and day 13 **l** (*n* = 3 mice each, 3 fields per tissue section); scale bar = 25 µm. White arrowheads indicate GFP^+^ cells. White asterisk denotes associated Supplementary Movie 1. Data represent mean ± s.e.m. *p< 0.05, **p<0.01,***p<0.001 (Statistical analysis by one-way ANOVA in g and *t*-test in **f**, **i** and **j**). Source data are provided as a Source Data file

We then investigated the fate of PDGFRα^+^ stromal progenitors in the mammary gland by crossing *Pdgfrα*^*Cre*^ mice to *Rosa26*^*mTmG*^ reporters. The resulting *Pdgfrα*^*Cre*^*R26*^*mTmG*^ lineage tracing mice have indelible eGFP labeling of *Pdgfrα*^+^ cells and their progeny after Cre-mediated excision of tdTomato in *Pdgfrα*-expressing cells (Fig. 2a). In adult *Pdgfrα*^*Cre*^*R26*^*mTmG*^ females, native GFP fluorescence was seen in adipocytes of mammary whole mounts by confocal imaging (Fig. 2b), confirming *Pdgfrα*^+^ cells as adipocyte precursors in vivo as previously reported for male adipose tissue depots^25^. Remarkably, GFP was observed throughout the gland in mammary epithelial structures (Fig. 2b and Supplementary Movie 1). Tissue sections revealed the distribution patterns of PDGFRα^+^ cells in relation to GFP^+^ progeny. Specifically, PDGFRα^+^ cells were dispersed in the fat pad and adjacent to ducts, yet restricted to stroma while GFP^+^ cells comprised PDGFRα^+^ stromal cells, adipocytes and epithelial cells (Fig. 2c, d and Supplementary Fig. 2a). GFP^+^PDGFRα^-^ cells were found in both the basal and luminal epithelial layers, with luminal GFP^+^ cells being primarily PR^-^ (Supplementary Fig. 2a). FACS enumeration demonstrated that GFP^+^ progeny was present in epithelial compartments in addition to stroma, and dominated the basal compartment and Sca1^-^ luminal progenitor subset, both of which are generally hormone-receptor negative (Fig. 2e, f and Supplementary Fig. 2b).

Given this unexpected occurrence of PDGFRα^+^ stromal progeny in epithelia, we performed ddPCR on FACS-purified mammary subsets from *Cre*^*+*^ mice and *Cre*^*-*^ controls to measure *Pdgfrα* expression in our lineage tracing study; *Pdgfrα* transcript was only detected in stroma (Fig. 2g). We next investigated PDGFRα^+^ progenitor dynamics during early development, and found that PDGFRα remained stromally-restricted while GFP^+^ cells in lineage tracing experiments were observed in epithelia of both pubescent (5-week) and prepubescent (2-week) glands through imaging and FACS analyses (Fig. 2h–j and Supplementary Fig. 3a-d). Tracing in the mammary rudiment at embryonic day 18 (E18) and bud as early as E13 showed localization of PDGFRα to the mammary mesenchyme and the presence of GFP progeny in epithelial layers that expressed EpCAM, K14 and K19 (Fig. 2k, l and Supplementary Fig. 3e, f). These in situ and quantitative data pinpointed PDGFRα^+^ stromal precursors as a previously unrecognized source of mammary epithelial progenitors.

### Adipocyte progenitors contribute to mammary epithelia

**Fig. 3:**
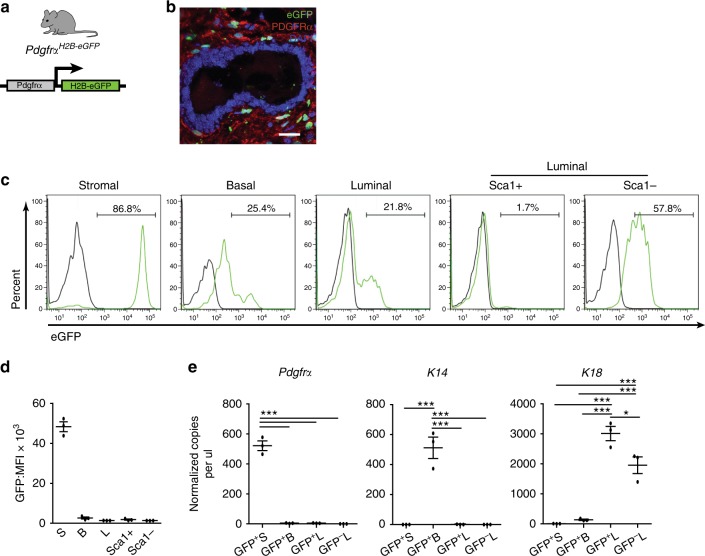
Endogenous Pdgfrα promoter activity in the mammary gland. **a** A mouse reporter for endogenous Pdgfrα promoter activity. **b** Co-staining for H2B-eGFP and PDGFRα in the adult gland (representative of *n* = 3 mice and 5 fields per tissue section); scale bar = 25 µm. **c** Flow cytometry histograms depicting H2B-eGFP^+^ cells (green) in mammary subsets relative to wild-type control (black). **d** Mean fluorescence intensity (MFI) of H2B-eGFP in (**c**) (*n* = 3 mice). **e** Droplet digital PCR analysis of *Pdgfrα*, *K14*, *K18* normalized to *Gapdh* in FACS-sorted GFP^+^ and GFP^-^ cell fractions from reporter mice (isolated from *n* = 3 individual mice). Data represent mean ± s.e.m. *p< 0.05, ***p<0.001 (one-way ANOVA). Source data are provided as a Source Data file

**Fig. 4:**
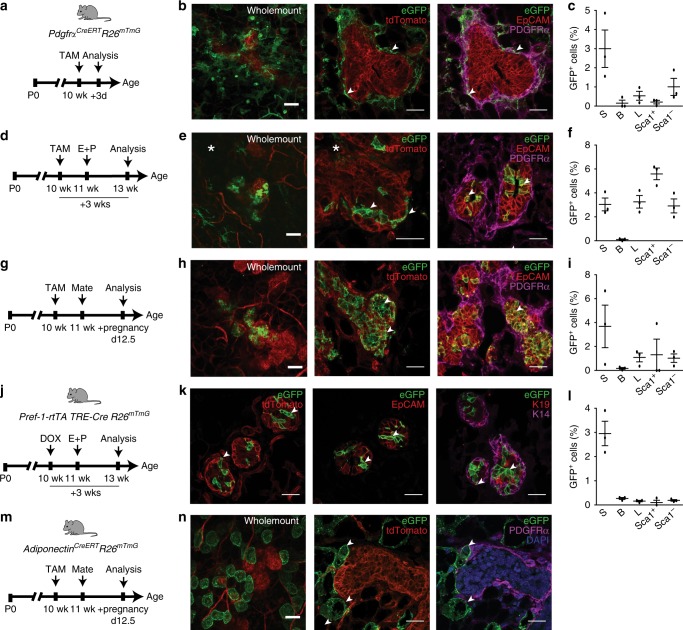
Mesenchymal adipocyte progenitors are recruited into the adult mammary epithelium during cellular expansion. **a** Short trace regimen in the tamoxifen (TAM)-inducible *PdgfrαCreERT R26mTmG* model. **b** Wholemount (left) and tissue sections immunostained for GFP (green), tdTomato (red) (middle) and GFP with EpCAM (red), PDGFRα (magenta) (right) isolated from an adult gland 3 days following TAM injection. **c** Flow cytometry quantification of GFP^+^ cells within stromal (S), basal (B), luminal (L), and luminal Sca1^+^, Sca1^-^ subsets in the 3d short trace (*n* = 3 mice). **d** Three week trace of PDGFRα^+^ cell progeny after TAM induction and hormone stimulation. **e** Native fluorescence within lobuloalveoli after E+P treatment (left) and GFP^+^ cells in tissue sections (middle, right). **f** GFP^+^ cells in mammary subsets after a 3 week trace (*n* = 3 mice). **g** PDGFRα^+^ cell tracing during pregnancy. **h** Wholemount fluorescence showing GFP^+^ lobuloalveolar structures during mid-pregnancy (left) and immunostaining on tissue sections (middle, right). **i** GFP^+^ cell proportions during pregnancy (*n* = 3 mice). **j** Lineage tracing Pref-1^+^ mesenchymal adipocyte progenitors in the *Pref-1-rtTA TRE-Cre R26mTmG* model following DOX induction and hormone stimulation. **k** Mammary tissue sections from Pref-1 tracing mice co-stained for eGFP (green) and tdTomato (red) (left), eGFP with epithelial markers EpCAM (red) (middle) and K19 (red), K14 (magenta) (right). **l** GFP^+^ cell distribution in mammary subsets of Pref-1 adult mice stimulated with hormones (*n* = 3 mice). **m** Mature adipocyte lineage tracing model subjected to TAM induction and pregnancy. **n** Mammary tissue isolated from adipocyte tracing mice analyzed by wholemount (left) and immunostaining (middle, right) with eGFP (green), tdTomato (red), PDGFRα^+^(magenta) and DAPI (blue), showing an absence of mature adipocyte recruitment into the epithelium (*n* = 3 mice). Scale bar = 50 µm for wholemounts and 25 µm for tissue sections; arrowheads indicate GFP^+^ cells. Images are representative of 5 fields per tissue section per mouse. White asterisk denotes associated Supplementary Movies 2 and 3. Data represent mean ± s.e.m. Source data are provided as a Source Data file

We next used a series of mouse models to discern the recruitment of mesenchymal stromal lineage cells into the adult mammary epithelium. We first checked endogenous *Pdgfrα* expression using *Pdgfrα*^*H2B-eGFP*^ mice that harbor a histone H2B-eGFP fusion reporter controlled by the *Pdgfrα* promoter (Fig. 3a). In immunostained tissue sections, GFP^+^ cells were present in the PDGFRα^+^ stroma but not epithelium (Fig. 3b). Flow cytometry showed a marked H2B-eGFP^+^ stromal population whereas epithelial cells expressed 1–2 orders of magnitude lower GFP fluorescence intensity (Fig. 3c, d). *Pdgfrα* analysis by ddPCR in FACS-purified GFP^+^ stroma versus low intensity GFP^+^ basal and luminal subsets verified detectable transcripts only in GFP^+^ stroma (Fig. 3e), indicating that the low epithelial GFP reflects prior *Pdgfrα* expression in the stromal parental lineage. We employed inducible *Pdgfrα*^*CreERT*^*R26*^*mTmG*^ mice which have previously been used to trace the adipocyte lineage in white adipose tissue^24^. Here, Cre-driven eGFP expression is induced following tamoxifen (TAM) administration (Supplementary Fig. 4a), enabling temporal control over cell labeling and fate mapping. In a 3-day short trace, mammary tissue from TAM-treated mice had prominent GFP in PDGFRα^+^ stromal cells (Fig. 4a–c and Supplementary Fig. 4b, e). Occasional GFP^+^ PDGFRα^-^ epithelial cells were noted, some of which had finger-like projections in contact with the PDGFRα^+^ stroma, signifying a transitory state (Supplementary Fig. 4c**)**. Oil-injected Cre^+^ controls did not show GFP in whole mounts, tissue sections or by flow cytometry, thus precluding possible leaky Cre expression (Supplementary Fig. 4d, e).

**Fig. 5:**
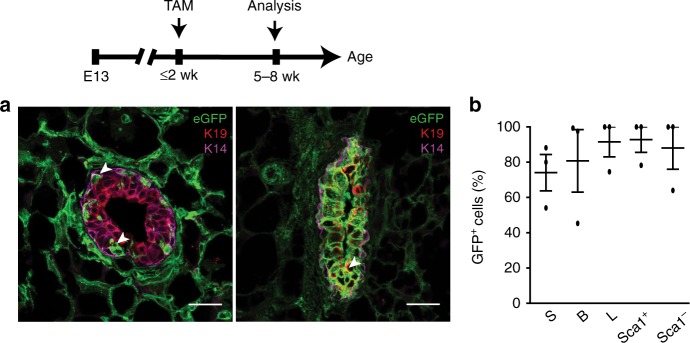
PDGFRα^+^ cell descendents contribute to early mammary epithelial development. **a** Immunofluorescent images of GFP-labeled cells with epithelial Keratins (luminal-K19, basal-K14) in 5–8 week old mammary tissue following TAM induction in prepubescent mice (representative of intermediate (left) and high (right) GFP labeling within the epithelium); *n* = 3 mice; scale bar = 25 µm. **b** GFP^+^ cells in stromal (S), basal (B), luminal (L), and luminal Sca1^+^, Sca1^-^ mammary subsets (*n* = 3 mice). Data represent mean ± s.e.m. Source data are available as a Source Data file

Ovarian sex hormones, especially progesterone, are well known to activate mammary stem cells and progenitors in the adult gland propelling the growth of milk-secreting lobuloalveoli during the mouse and human reproductive cycle as well as pregnancy^1,10,32,33^. Thus, we incorporated sex hormone treatment or pregnancy in a ~3-week trace following TAM (Fig. 4d–i). After hormone exposure, GFP^+^ cells contributed to epithelial expansion in lobuloalveolar structures, remained PDGFRα^-^, were largely PR^-^ and epithelial EpCAM^+^ although Keratin low, and accumulated mainly in luminal progenitor subsets (Fig. 4e, f and Supplementary Fig. 5a, b and Supplementary Movie 2, 3). Of note, PDGFRα^+^ GFP^+^ cells often exhibited long cellular processes, and in certain instances formed long stretches contiguous with PDGFRα^-^ GFP^+^ cells in the epithelium (Fig. 4e and Supplementary Movie 3). Tracing in mid-pregnancy (Fig. 4g), GFP-labeled cells similarly demonstrated their expansion within lobuloalveoli, again mostly in the luminal compartment (Fig. 4h, i and Supplementary Fig. 5c, d). We also found that induction of lineage tracing during early development in mice 2 weeks old or less resulted in a vast proportion of GFP^+^ progeny in luminal as well as basal subsets when analyzed in older mice, indicating significant contribution of PDGFRα^+^ cells to early formation of the mammary gland (Fig. 5a, b and Supplementary Fig. 6).

Preadipocyte factor-1 (Pref-1 or Dlk1) has been shown to specifically mark mesenchymal adipocyte precursors essential during embryonic white adipose tissue development and adult adipose tissue expansion^34,35^. To ascertain the contribution of mesenchymal adipocyte precursors to the mammary epithelium, we selected doxycyline (DOX)-inducible *Pref-1-rtTA TRE-Cre R26*^*mTmG*^ mice as an independent lineage tracing model (Fig. 4j). In a short 3-day trace following DOX administration in adult mice, GFP-labeled cells were present in mammary stroma surrounding ducts where some overlapped with PDGFRα expression (Supplementary Fig. 7a). Upon longer-tracing involving hormone-stimulated growth of the mammary gland, GFP^+^ cells were observed within mammary lobuloalveoli and epithelial subsets (Fig. 4k, l and Supplementary Fig. 7b, c), similar to *Pdgfrα*^*CreERT*^*R26*^*mTmG*^ mice. To determine whether mature adipocytes have the capacity to generate mammary epithelial cells, we TAM-induced adult *Adiponectin*^*CreERT*^*R26*^*mTmG*^ mice which have been shown to effectively label mature adipocytes^36^ (Fig. 4m). Tracing cell fate at mid-pregnancy showed GFP-labeled adipocytes without any epithelial progeny (Fig. 4n and Supplementary Fig. 7d, e). GFP^+^ adipocytes were also negative for PDGFRα expression (Fig. 4n), as reported previously^25^ . Together, these data show that mesenchymal adipocyte precursors but not mature adipocytes within the mammary stroma give rise to epithelial descendents which expand during hormone-dependent growth of the adult mammary gland.

### Tracing-in-transplants illustrate stromal-to-epithelial transition

**Fig. 6:**
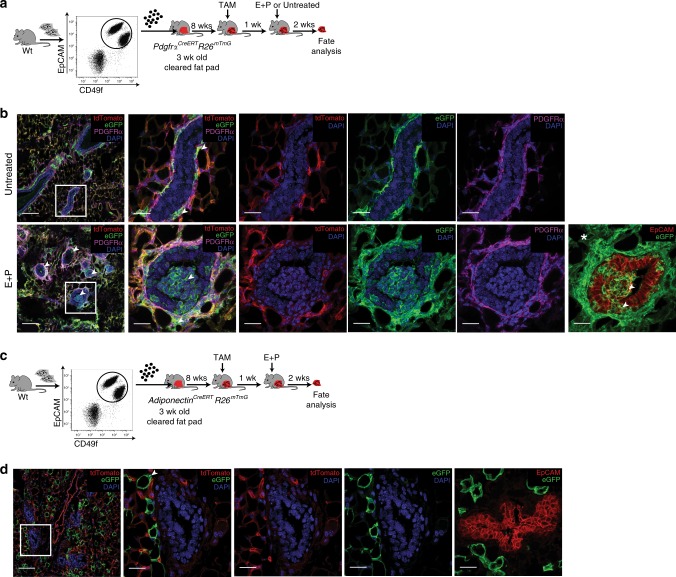
Stromal to epithelial transition of mesenchymal cells in a tracing-in-transplant assay. **a** Stromal cell contribution to epithelia was tested by a lineage tracing-in-transplant strategy utilizing FACS-sorted wild type non-fluorescent epithelial cells which were injected into inguinal cleared fat pads of *PdgfrαCreERT R26mTmG* tracing mice that were later TAM induced and left untreated (*n* = 6 fat pads) or treated with E+P hormones (*n* = 6 fat pads). **b** Images of outgrowths representative of all transplanted fat pads (white boxed area on leftmost images shown at higher magnification on right panels) with tdTomato (red), GFP (green), PDGFRα (magenta), DAPI (blue) and EpCAM (red). **c** Sorted wild type epithelial cell injections into cleared fat pads of *AdiponectinCreERT R26mTmG* mature adipocyte lineage tracing mice, also exposed to sex hormones following TAM induction. **d** Representative outgrowths (*n* = 5) resulting from transplants in (**c**). Scale bar = 100 µm (left; low magnification) and 25 µm (close-ups).; arrowheads indicate GFP^+^ cells. White asterisk denotes associated Supplementary Movie 4

We then designed a lineage ‘tracing-in-transplant’ assay (Fig. 6a) to delineate whether mesenchymal cells from a stromal lineage tracing fat pad can indeed contribute to epithelium derived from a non-lineage tracing mouse. FACS-purified, non-fluorescent wild-type mammary epithelial cells were transplanted into inguinal epithelium-divested mammary fat pads of *Pdgfrα*^*CreERT*^*R26*^*mTmG*^ recipients; care was taken to sever the connection between the fourth and fifth mammary glands to ensure complete clearing of the fourth mammary fat pad. Recipients received TAM 8-weeks after transplant, and then were hormone-stimulated or left untreated. In all transplants, wild type outgrowths were readily visible in tissue sections as nuclear DAPI^+^ and tdTomato^-^ in contrast to the endogenous DAPI^+^ tdTomato^+^ lineage tracing fat pad (Fig. 6b). Notably, sex hormone stimulation following TAM induction generated wild-type outgrowths in all transplanted fat pads that contained GFP^+^ progeny from the stroma that now contributed to mammary epithelial structures, lost their mesenchymal PDGFRα expression, and instead expressed the epithelial marker EpCAM (Fig. 6b and Supplementary Movie 4). GFP^+^EpCAM^+^ epithelial cells also existed as clusters, indicating expansion of epithelial progenitors derived from stromal precursors. In mice untreated with hormones, GFP-labeled cells were only detectable in the stroma (Fig. 6b), indicating that the transplantation technique per se did not elicit a stromal to epithelial lineage switch. Furthermore, when wild type epithelial cells were transplanted into mature adipocyte lineage tracing fat pads of *Adiponectin*^*CreERT*^*R26*^*mTmG*^ mice that were subsequently TAM induced and hormone treated, GFP-labeled adipocytes in the stroma did not contribute to epithelia (Fig. 6c, d), showing that sex hormones do not mediate artificial epithelial recruitment of otherwise committed stromal lineages. These series of transplantation experiments unequivocally demonstrate the transition of a mesenchymal niche population into mammary epithelial progenitors.

### Adipocyte progenitors exhibit a migratory phenotype

**Fig. 7:**
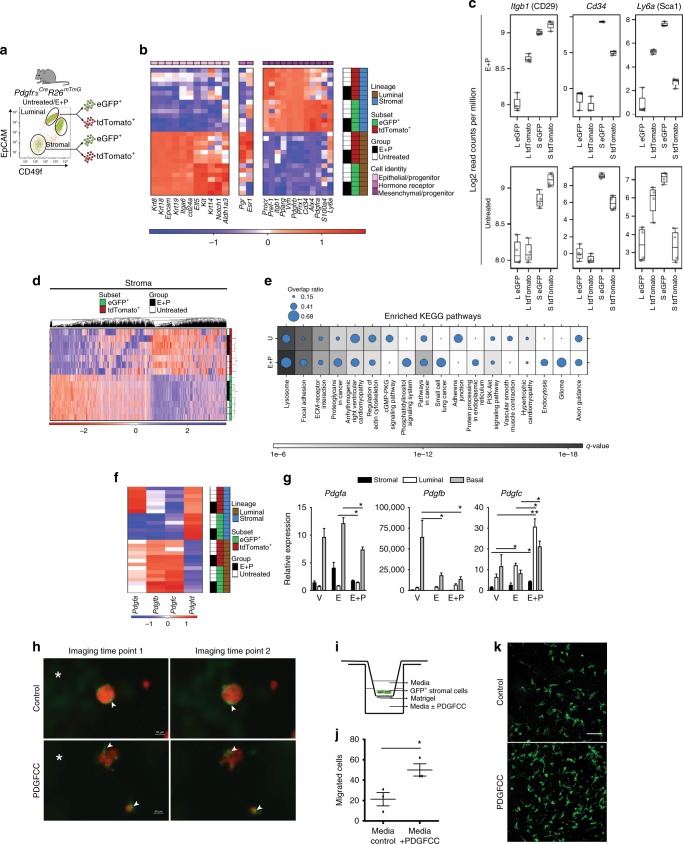
Stromal progenitors migrate into the mammary epithelium in response to hormone-driven signals. **a** Sorted samples from untreated (*n* = 4) and E+P treated (*n* = 3) *PdgfrαCre R26mTmG* adult mice for RNA-seq. **b** Cellular identity of luminal GFP^+^, tdTomato^+^ and stromal GFP^+^, tdTomato^+^ mammary cells. **c** Read counts per million for adipocyte progenitor genes in indicated mammary subsets. Boxplots depict the upper and lower quartiles, with the median shown as a solid line; whiskers indicate 1.5 times the interquartile range. **d** Heat map showing clustering of the top 20% most variable genes in all stromal samples. **e** Top 10% most significantly enriched pathways for DEGs between GFP^+^ and tdTomato^+^ stromal cells from untreated (U) and E+P-stimulated mice (dot size = # of DEGs / # of genes in pathway). **f** Heat map of PDGFR ligand (*Pdgf*) expression by RNA-seq in sorted luminal and stromal samples. **g** qRT-PCR analysis of *Pdgf* expression relative to β-actin in FACS-sorted subsets isolated from ovariectomized mice treated with vehicle (V), E or E+P (*n* = 3 mice per group). **h** Live imaging of FACS-sorted GFP^+^ stromal cells from adult *PdgfrαCre R26mTmG* mammary glands co-cultured with tdTomato^+^ organoids derived from sorted epithelial cells of Cre^-^ mice, and left untreated (control) or treated with PDGFCC and imaged over night for ~12 h; scale bar = 50 µm. **i** Modified Boyden chamber assay used to analyze migration of FACS-sorted GFP^+^ stromal cells from *PdgfrαCre R26mTmG* mice in response to PDGFCC. **j** Quantification of migrated cells in **i** (*n* = 3 technical replicates). **k** Representative images of migrated GFP^+^ stromal cells; scale bar = 100 µm. White asterisk denotes associated Supplementary Movies 5 and 6. Data represent mean ± s.e.m. *p< 0.05, **p<0.01 (one-way ANOVA). Source data are provided as a Source Data file

To define molecular features of adipocyte progenitors and their epithelial progeny, we performed RNA-seq on FACS-purified GFP^+^ and tdTomato^+^ stromal and luminal populations from untreated (*n* = 4) and hormone stimulated (*n* = 3) adult *Pdgfrα*^*Cre*^*R26*^*mTmG*^ mice (Fig. 7a). The mRNA abundance of known lineage markers confirmed a mesenchymal phenotype for the stromal subsets (*e.g. Vim*, *S100a4*, *Procr*, as well as adipocyte precursor markers *Pdgfra*, *Pref-1 (Dlk1)*, *Pparg*) while the luminal fractions showed high levels of epithelial markers (*e.g. Krt8, K18, K19, Epcam, Itga6* along with basal *Krt14;* Fig. 7b). GFP^+^ luminal cells had higher levels of epithelial progenitor genes such as *Elf5*, *Kit*, *Notch1* and *Aldh1a3* and lower levels of estrogen (*Esr1*) and progesterone (*Pgr*) receptors, thus signifying enrichment of progenitor features within the GFP^+^ epithelial progeny. Previous studies have assigned a CD29^+^CD34^+^Sca1^+^ profile to adipocyte progenitors^23,24^. CD29 and Sca1 are also used in the mammary gland field to distinguish epithelial subpopulations, although we noted *Itgb1* (CD29) to be higher in stromal subsets, and *Ly6a* (Sca1) and *Cd34* to be elevated in the GFP^+^ stromal subpopulation (Fig. 7c), confirming their adipocyte precursor phenotype. *Alx4*, a stromally restricted homeodomain factor essential for mammary morphogenesis^37^, was also higher in the GFP^+^ adipocyte progenitor population that expressed *Pdgfrα*, where *Alx4* increased in response to hormones. These expression profiles affirmed the distinct nature of mesenchymal and epithelial subpopulations purified for our molecular analyses.

Transcriptome comparison of GFP^+^*vs*. tdTomato^+^ populations from all stromal specimens identified 7555 statistically significant differentially expressed genes (DEGs; FDR < 0.01). When the top 20% most variable genes were examined by unsupervised hierarchical clustering, stromal GFP^+^ and tdTomato^+^ subsets clustered into discrete groups (Fig. 7d). GFP^+^ stromal cells were observed to have more similar transcriptomes to luminal cells than to tdTomato^+^ stromal cells following distance-analysis of DEGs between GFP^+^ and tdTomato^+^ subpopulations within stromal and luminal lineages (Supplementary Fig. 8a, b). KEGG pathway analysis of DEGs between GFP^+^ and tdTomato^+^ stromal subsets indicated enrichment in pathways that govern Focal adhesion, ECM-receptor interaction, Actin cytoskeleton, Lysosomes and PI3K-Akt signaling (Fig. 7e). Exposure to hormones augmented pathways in cancer, as well as those implicated in cell migration such as Focal Adhesion, Endocytosis and the Phosphatidylinositol System^38–40^. We also noted a decrease in Adherens junction. Interestingly, PDGFRα and its downstream PI3K pathway are involved in directed cell migration^26^. These analyses inferred a cell migration program is heightened within stromal progenitors following their activation.

**Fig. 8:**
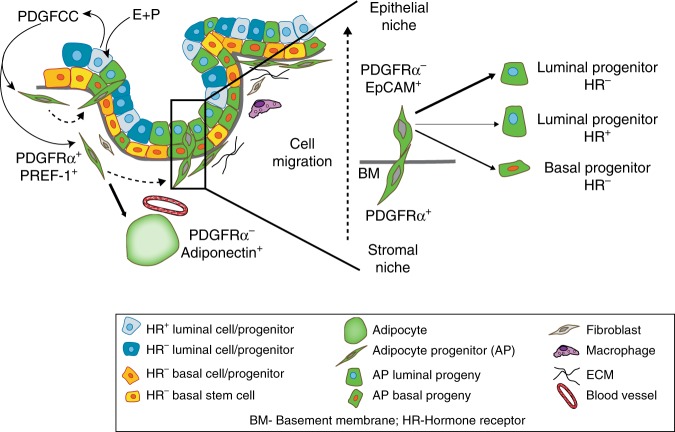
Model illustrating the plasticity of stromal progenitors in the mammary gland. The mammary stroma is home to PDGFRα^+^ PREF-1^+^ mesenchymal progenitor cells that are found in close promixity to the parenchymal epithelium. These cells can differentiate into adipocytes (Adiponectin^+^) in the mammary fat pad, or switch to an epithelial cell fate (EpCAM^+^). To undergo this stromal-to-epithelial transition, mammary epithelial cells are stimulated by adult sex hormones such as estrogen and progesterone (E+P) to secrete PDGFCC, a PDGFRα ligand, which induces chemotactic migration of PDGFRα^+^ mesenchymal progenitors into the epithelial niche. In the epithelium, these stroma-derived cells generate epithelial progeny, primarily giving rise to the hormone receptor (HR)- negative luminal lineage in the adult during epithelial expansion

We postulated that PDGFRα ligands expressed by epithelial cells in response to hormones could serve as chemotactic stimuli for stromal cell migration. Interrogation of RNA-seq data for known PDGFR ligands revealed that *Pdgfa*, *Pdgfb* and *Pdgfc* were expressed by epithelial cells while *Pdgfd* was mostly present in stroma (Fig. 7f). Upon hormone exposure, especially in response to both estrogen and progesterone, *Pdgfc* was most significantly elevated in luminal and basal epithelial subsets, as seen by qRT-PCR of FACS-purified stromal and epithelial fractions (Fig. 7g). In co-cultures of FACS-sorted GFP^+^ stromal cells from *Pdgfrα*^*Cre*^*R26*^*mTmG*^ mice with Cre^-^ tdTomato^+^ mammary epithelial organoids, addition of PDGFCC was seen to stimulate GFP^+^ stromal cell movements into epithelial organoids as monitored through live time-lapse imaging (Fig. 7h and Supplementary Movie 5, 6). To determine whether PDGFCC stimulates chemotactic migration of GFP^+^ stromal cells that generate epithelial progeny, the FACS-sorted cells above were plated onto transwell inserts in a Boyden chamber assay, and cell migration measured in response to recombinant PDGFCC (Fig. 7i). This PDGFRα ligand significantly increased stromal cell migration compared to control (Fig. 7j, k). Thus, adipocyte precursor cells in the mammary stroma are likely activated in response to expansion cues through a hormone-PDGFCC-PDGFRα axis to disengage from their adipogenic program, migrate into the epithelium and adopt an epithelial cell fate (Fig. 8).

## Discussion

Our findings show plasticity of mesenchymal cells within stroma wherein adipocyte progenitors undergo cell fate transformation into epithelial progenitors during mammary parenchymal growth. Previous work has documented a potential of in vitro expanded adipose derived stromal cells to reprogram into different cell types primarily in in vitro settings^41–43^, where the stromal cells comprise of a heterogeneous population derived from the adipose stromal-vascular fraction and lack specificity for adipocyte progenitors. A previous report described a capacity of mature adipocytes to transdifferentiate into mammary secretory epithelial cells during pregnancy, and of secretory epithelial cells to revert to adipocytes during involution^44^. In contrast, we did not observe adipocyte to epithelial transformation with the mature adipocyte-specific Adiponectin-CreERT line. Interestingly, a recent study found that metastasis formation could be suppressed when invasive breast cancer cells that have undergone an epithelial to mesenchymal transition are induced to become adipocytes^45^, underscoring the highly plastic nature of mesenchymal cells.

Adipose tissue also plays an important role in energy homeostasis and excess adiposity is linked to increased risk of epithelial cancers including breast cancer^46,47^. In addition to adipocyte hypertrophy, adipose tissue accumulation is a result of adipocyte hyperplasia that is attributed to a recruitment of adipocyte progenitors^48^. Several mechanisms have been proposed to underlie the pathophysiological link between adiposity and cancer, largely involving adipokines and insulin^49^. Since most adiposity-related cancers occur in organs that are embedded in adipose tissue such as the breast, the roles of local adipose tissue resident cells including their progenitors in cancer development and progression are also of great interest^50^. Thus, studying adipocyte progenitor biology in the normal breast is essential for understanding its potential involvement in breast cancer.

In this study, we add a fundamental layer to the mammary epithelial hierarchy where stromal cells that possess an adipocyte progenitor identity exit their mesenchymal niche to populate the epithelial niche and actively generate cells within progenitor-enriched compartments, culminating in epithelial expansion during mammary growth. Postnatal mammary growth and remodeling are tightly controlled by hormones^1^ and we observed stromal cell recruitment and increased presence of their progeny in the epithelium during hormone-driven stages. Exogenous sex hormone supplementation in many of our experiments induced a consistent growth stimulus that mirrors in part the hormone milieu found during the physiological progesterone-dominant reproductive phase and pregnancy, resulting in lobuloalveolar formation. Further, we report PDGFRα^+^ mesenchymal cell contribution to the mammary epithelial network during early development and pregnancy. We provide evidence that steroid sex hormones induce PDGF signals in the epithelium which act to mobilize PDGFRα^+^ mesenchymal niche cells, stimulating their migration in a chemotactic manner. In the human breast, a distinct fibroblast lineage which has adipogenic capacity and supports the growth of luminal epithelial progenitors has been reported that accumulates around terminal ductal lobular units (TDLUs)^51^. It is possible that this fibroblast lineage has properties similar to the mesenchymal population that we describe here given that we see a prominent contribution of mesenchymal adipocyte progenitors to mouse mammary lobuloalveoli which are analogous structures to human TDLUs^52^. We show that this stromal lineage preferentially contributes to luminal progenitors in the adult mammary gland, a population implicated in aggressive *BRCA1*-mutated breast cancer^53,54^. Increased lifetime hormone exposure is a well known risk factor for breast cancer^55^ and in combination with increased adiposity or an aberrant metabolic state, hormone-responsive adipocyte precursors may contribute to malignant transformation in breast epithelia. In light of the global health crisis that is underway with increasing rates of adiposity, diabetes and associated increased risk of several cancers^56^, deconstructing adipocyte progenitor biology within tissue niches is pivotal not only for understanding its impact on adipose tissue mass, but also for elucidating the link between adiposity and cancer.

## Methods

### Mice

*Pdgfrα*^*Cre*^ (Stock# 013148), *Pdgfrα*^*CreERT*^ (Stock# 018280), *Rosa26*^*mTmG*^ (Stock# 007676), *Pdgfrα*^*H2B-eGFP*^ (Stock# 007669), *Adiponectin*^*CreERT*^ (Stock# 024671) mice were obtained from the Jackson Laboratory. C57BL/6 mice were purchased from Charles River Laboratories. *Pref-1-rtTA TRE-Cre Rosa26*^*mTmG*^ mice were provided by Dr. Hei Sook Sul (UC Berkeley). All mice were on the C57BL/6 background and cared for according to the Canadian Council for Animal Care guidelines under protocols approved by the Animal Care Committee of the Princess Margaret Cancer Centre, Toronto, Ontario.

### Treatments and lineage tracing

Mice were randomly assigned to groups that either received hormone treatment or were left untreated. Adult wild-type female mice (10–13 weeks old) were treated with 17β-estradiol (E, 0.14 mg) or 17β-estradiol + progesterone (E+P, 0.14 mg E+ 14 mg P) using 14-day slow release pellets (Innovative Research of America) implanted subcutaneously. Hormone treatments were performed in intact mice unless otherwise indicated in the figure legend. Lineage tracing in the *Pdgfrα*^*Cre*^*R26*^*mTmG*^ constitutive model was performed in adult (10–12 week old; comparable estrous stage), pubescent (5 week old), prepubescent (2 week old) female mice and in E18, E13 female embryos. Mice were randomly taken from different litters. For inducible lineage tracing in *Pdgfrα*^*CreERT*^*R26*^*mTmG*^ and *Adiponectin*^*CreERT*^*R26*^*mTmG*^ adult (10 week old) mice, a single intraperitoneal injection of 1.25 mg of 4-hyroxytamoxifen (TAM; Sigma) diluted in sunflower seed oil per 25 g body weight was administered to induce Cre-mediated recombination and GFP labeling. Lineage tracing in *Pdgfrα*^*CreERT*^*R26*^*mTmG*^ mice less than or equal to 2 weeks of age was induced with 0.5 mg of TAM. Lineage tracing in adult *Pref-1-rtTA TRE-Cre Rosa26*^*mTmG*^ mice was induced with a single intraperitoneal injection of 2 mg doxycycline (Sigma) in sterile PBS.

### Mammary cell preparation and flow cytometry

Freshly dissected mammary glands from individual mice were digested for 2.5 h at 37 °C in DMEM: F12 with 750 U ml^−1^ collagenase and 250 U ml^−1^ hyaluronidase. Digested tissue was vortexed, incubated in NH_4_Cl red blood cell lysis solution, dissociated in 0.25% trypsin for 2 min and 5mg ml^−1^ dispase with 0.1mg ml^−1^ DNaseI for 2 min, prior to filtering through a 40μm mesh to obtain single cells. All reagents were from STEMCELL Technologies. Cells were then labeled with fluorochrome (Pe-Cy7)-conjugated antibodies to CD31 (Ebioscience, Cat.# 25–0311, 1:500), Ter119 (Ebioscience, Cat.# 25–5921, 1:200) and CD45 (Ebioscience; Cat.# 25–0451, 1:1000) for endothelial, erythrocyte and hematopoietic cell exclusion respectively. For segregating mammary epithelial and stromal subpopulations, antibodies used are as follows: anti-CD49f-FITC (BD Pharmingen, Cat.# 555735, 1:50) or anti-CD49f-APC (R&D, Cat.# FAB13501A, 1:50), anti-EpCAM-AF647 (Biolegend, Cat.# 118211, 1:200) or anti-EpCAM-APC/Cy7, Cat.# 118217, 1:200), anti-Sca1-APC/Cy7 (Biolegend, Cat.# 108125, 1:500) or anti-Sca1-Pacific Blue (Biolegend, Cat.# 108119, 1:500) and anti-CD140 (PDGFRα)-PE (Biolegend, Cat.# 135905, 1:200). Dead cells were excluded with propidium iodide staining (PI, Sigma). Flow cytometry analysis was done using FACSCantoII (BD) and FlowJo software (Tree Star, Inc.). Cell sorting was performed on a FACSAria (BD) and the purity of sorted populations was routinely >96%.

### Colony forming cell assay and adipocyte differentiation

Stromal mammary subpopulations were FACS-sorted and plated with irradiated NIH 3T3 fibroblasts in DMEM:F12 medium containing 10% FBS (GIBCO), insulin (Life Technologies), EGF (STEMCELL Technologies), cholera toxin (Sigma), adenine (Sigma), hydrocortisone (STEMCELL Technologies), Rock inhibitor (Reagents Direct) and cultured in 5% oxygen conditions as done previously for mammary epithelial cells^11^. Colonies were scored after 10 days. To induce adipocyte differentiation, the FACS-purified adipocyte progenitor subset was cultured in DMEM supplemented with 10% FBS (GIBCO) and 10 ng/ml bFGF (STEMCELL Technologies), as previously described^23^. Cells were grown to confluence and held at confluence for 2 days without media change following which a differentiation cocktail containing insulin, dexamethasone and IBMX was added in fresh media without bFGF for 3 days. Cells were then maintained in DMEM with 10% FBS for another 3 days, and subsequently fixed with 2% formaldehyde and 0.2% glutaraldehyde in PBS for 15 min. Cultures were rinsed carefully in PBS, water and 60% isopropanol prior to staining with oil red O (0.7% in 60% isopropanol) for 20 min at room temperature after which they were rinsed with 60% isopropanol and water.

### Tissue processing and immunostaining

Dissected mammary glands were fixed for 2 h in 4% paraformaldehyde at room temperature, washed three times with PBS and immersed in 30% sucrose in PBS overnight at 4 °C, after which they were embedded in OCT, stored at −80 and cryosectioned at 5–15μm thickness for immunofluorescence. Frozen sections were incubated in blocking buffer (5% normal donkey serum/1% BSA/0.2% Triton in PBS) for 1 h at room temperature followed by primary antibodies diluted in blocking buffer without Triton overnight at 4 °C. Sections were rinsed and incubated with secondary antibodies for 1 h at room temperature, rinsed again and mounted using ProLong Gold Anti-fade reagent with DAPI. For immunohistochemistry, 4% paraformaldehyde-fixed paraffin-embedded tissue sections were de-paraffinized in xylene, gradually rehydrated in descending concentrations of ethanol, and antigen retrieval performed in Borg Decloaker solution (pH 9) for 5 min at 125 °C using a Decloaking chamber (Biocare Medical). After rinsing in PBS, endogenous peroxidase, avidin and biotin blocking buffers were applied, followed by 5% horse serum block in PBS. Sections were incubated overnight in primary antibody at 4 °C, washed the next day in PBS and incubated with biotinylated horse anti-goat secondary (Vector Labs, Cat.# BA9500, 1:1000) for 1 h at room temperature, followed by Vectastain Elite ABC peroxidase, AEC substrate and counterstained with Mayer’s hematoxylin. Primary antibodies used are as follows: anti-PDGFRα (R&D systems, Cat.# AF1062, 1:200), anti-K8 (LifeSpan Biosciences, Cat.# LS-B12422, 1:200), anti-K14 (Biolegend, Cat.# 906001, 1:400 or Cat.# 905301, 1:1000), anti-K19 (Santa Cruz, Cat.# sc-33111, 1:100), anti-GFP (Abcam, Cat.# ab13970, 1:4,000), anti-tdTomato (Clontech, Cat.# 632496, 1:500), anti-PR (Santa Cruz, Cat.# sc-7208, 1:200) and anti-EpCAM (abcam, Cat.# ab71916, 1:100). Secondary antibodies used are as follows: anti-goat, anti-rabbit, anti-chicken conjugated to AlexaFluor 647, AlexaFluor Cy3, AlexaFluor 488 or Dylight 405 (Jackson ImmunoResearch, 1:300).

### Confocal microscopy image acquisition

Freshly dissected and unprocessed mammary glands were placed between a slide and coverslip to image as whole mounts on a Zeiss LSM710 confocal microscope using 20 × /1.0NA (water) and C-Apo 63 × /1.4NA (oil) objectives. Fat was left intact to capture adipocytes. Z-stacks were acquired at 1024 × 1024 optical section resolution. Tissue sections were imaged on a Zeiss LSM700 confocal microscope using Fluar 10×/0.50NA, Plan-Apochromat 40×/1.4NA and 60 × /1.4NA oil-immersion objective lenses. Imaging was performed on 3–5 fields per tissue section. Optical sections and Z-projections were analyzed for every tissue section. Image acquisition was performed with Zen and final image composites and maximum intensity Z-projections were generated with Zen software and ImageJ. Imaris was used for 3D rendering.

### Live cell imaging

FACS-purified GFP^+^ cells from the lin^-^ stromal fraction of *Pdgfrα*^*Cre*^*R26*^*mTmG*^ mammary tissue were co-cultured with sorted tdTomato^+^ epithelial cells from Cre^-^*R26*^*mTmG*^ mammary glands on top of a thin layer of phenol red-free matrigel (BD Biosciences) in a Lab-Tek II 4 chambered cover glass system (Nunc) in DMEM:F12 medium containing 5% FBS (GIBCO), insulin (Life Technologies), EGF (STEMCELL Technologies), bFGF (STEMCELL Technologies), hydrocortisone (STEMCELL Technologies), Rock inhibitor (Reagents Direct) in 5% oxygen conditions. After 4 days of organoid growth, cultures were treated with vehicle control (4 mM HCl + 0.1% BSA) or 100 ng/ml PDGFCC (R&D systems) and imaged overnight with Zen software using 20 × /0.8 Plan-Apochromat objective lens of an inverted motorized Zeiss AxioObserver microscope equipped with a stage top heated CO_2_ incubator.

### Cell migration assay

In a modified Boyden Chamber assay, *Pdgfrα*^*Cre*^*R26*^*mTmG*^ GFP^+^ stromal cells (40,000 cells/well) suspended in DMEM:F12 medium were placed on 6.5 mm transwell inserts with a 8.0 μm pore size and underside coated with 30μl of phenol red-free matrigel (BD Biociences) in 24 well plates (Corning). DMEM: F12 medium with vehicle control (4 mM HCl + 0.1% BSA) or 100 ng/ml PDGFCC (R&D systems) was added to the bottom chambers and cells incubated at 37 C for 4 h. Non-migrated cells were removed from the insert with a cotton swab, and migrated cells in the matrigel layer were fixed with paraformaldehyde and washed with PBS. GFP^+^ migrated cells were counted after imaging inserts on a cell imaging dish (Eppendorf) using a Zeiss LSM700 confocal microscope with Fluar 10X/0.50NA objective lens. Four microscope fields were counted per well and treatments performed in triplicate.

### Lineage tracing-in-transplants

Mammary epithelial cells were FACS-sorted from adult wild-type non-reporter female mice and transplanted into the fourth inguinal *Pdgfrα*^*CreERT*^*R26*^*mTmG*^ or *Adiponectin*^*CreERT*^*R26*^*mTmG*^ inducible lineage tracing mammary fat pads (10,000 cells per fat pad) after surgical severing of the fourth and fifth mammary gland connection and clearing of the endogenous mammary epithelium of 21 day old recipient females. After 8 weeks, recipient mice were injected once with TAM to induce Cre-mediated recombination and GFP labeling in the fat pad, rested for 1 week and then stimulated with hormones for 14 days or left untreated. Untreated mice were analyzed at the estrus phase. Resulting outgrowths were analyzed by confocal immunofluorescence on stained frozen sections of dissected mammary fat pads.

### qPCR and ddPCR

RNA was prepared from FACS-sorted primary mammary cell fractions using the PicoPure RNA Isolation Kit (Arcturus). During isolation, samples were also treated with RNase-free DNase (Qiagen) to remove any contaminating DNA. The quality and concentration of RNA was determined by analysis with a NanoDrop 2000 Spectrometer (260/280 ratio; Thermo Scientific). For RT-PCR studies, total RNA was reverse transcribed into first strand cDNA and amplified using the SMARTer PCR cDNA Synthesis Kit and Advantage2 PCR Kit (Clontech). Relative quantification Real-time PCR (ΔΔCt) was performed on 2 ng of amplified and purified cDNA using an ABI PRISM 7900HT Sequence Detection System (Applied Biosystems). TaqMan gene expression assay mix (Applied Biosystems) containing unlabeled PCR primers and FAM-labeled TaqMan MGB probes were used to detect expression of *Pdgfrα* (Assay ID: Mm00440701_m1), *Pdgfa* (Assay ID: Mm01205760_m1), *Pdgfb* (Assay ID: Mm00440677_m1), *Pdgfc* (Assay ID: Mm00480205_m1) and *β-actin* (*ACTB*, Assay ID: Mm01205647_g1). Data were analyzed using Sequence Detection System software (v2.3; Applied Biosystems). The threshold cycle (C_T_) values were used to calculate relative RNA expression levels. Transcript levels were normalized to endogenous β-actin transcripts and compared to vehicle control stromal expression set at 1. First strand cDNA for droplet digital PCR (ddPCR) was prepared using qScript cDNA supermix (QuantaBio). The Bio-Rad QX200 droplet generator was used to generate 20μl of droplets, using the Bio-Rad protocol, from a mix of 5 ng cDNA, 2× ddPCR supermix (Bio-Rad), forward and reverse primers (450 nM), probe (FAM or HEX, 200 nM) and deionised distilled water (to 25μl). Droplets were cycled in a Bio-Rad C1000 at 95 °C for 10 min; 50× (94 °C −30 s, 61 °C − 60 s); 98 °C for 10 min, then 4 °C until analysis on the QX200 droplet reader using the QuantaSoft v1.4.0 software provided by Bio-Rad. Primers and probes for ddPCR are shown in Supplementary Table 1. All oligos were from Eurofins Genomics and all probes were from Integrated DNA Technologies.

### RNA sequencing

RNA was isolated from FACS-sorted eGFP^+^ and tdTomato^+^ subsets from stromal and luminal mammary populations of individual mice using the PicoPure RNA Isolation Kit (Arcturus). RNA concentrations and RNA quality were measured using a bioanalyzer (Agilent) (0.095–193 ng/μl; RIN 1–10). cDNA was generated from 2 ng of RNA with the SMARTer stranded total RNA-seq kit – pico input mammalian (Takara) using a modified fragmentation time (0–4 min) to accommodate lower quality RNA. cDNA fragments were indexed and amplified before being cleaned up (Beckman) and depleted of ribosomal RNA (Takara). Following depletion, cDNA libraries were amplified over 15 cycles of PCR. cDNA libraries were sequenced on a NextSeq 500 system (Illumina) in paired-end 75 mode.

### RNA-seq analysis

Alignment of raw sequence reads to the *Mus musculus* GRCm38.p5 GENCODE reference genome and the generation of gene level abundance estimates were performed using the STAR aligner (v.2.5.2a)^57^. Multivariate linear regression analysis was accomplished using the R package limma (v.3.32.2)^58^ and genes with Benjamini-Hochberg corrected *P* values less than 0.01 were considered as differentially expressed. KEGG pathway analysis was performed using the R package gprofiler (v.0.6.1)^59^ and top 10% most significantly enriched pathways were selected for comparison. Plots were generated using the BPG R package (v.5.7.1). All analyses and visualization were run in the R statistical environment (v.3.4.0).

### Statistical analyses

Sample sizes were chosen based on previous experience with comparable experiments utilizing animal models that generated measurable and reproducible responses^10,11^. In every instance, n represents a distinct biological replicate unless otherwise stated. Data for biological replicates were acquired on the same day or pooled from independent days. Where possible, experiments were repeated at least two independent times with successful replication. Data were analyzed using GraphPad prism software and reported as mean ± standard error of the mean (s.e.m). Comparison of data between multiple groups was performed using one-way analysis of variance (ANOVA) followed by Tukey’s post-hoc multiple comparison test, and analysis between two groups was made using Student’s *t*-test (two-tailed). Statistical significance is recognized at *p*<0.05.

### Reporting summary

Further information on research design is available in the Nature Research Reporting Summary linked to this article.

## Supplementary information

Supplementary Information

Description of Additional Supplementary Files

Supplementary Movie 1

Supplementary Movie 2

Supplementary Movie 3

Supplementary Movie 4

Supplementary Movie 5

Supplementary Movie 6

Peer Review File

Reporting Summary

Source Data

## Data Availability

Data that support the findings of this study are available from the corresponding author upon reasonable request. RNA-sequencing data are available from GEO under accession number GSE123714. The source data underlying Figs. 1b, e, i, 2f, g, i, j, 3d, e, 4c, f, i, l, 5b, 7b-g, j and Supplementary Figs. 1b and 8a, b are provided as a Source Data file.

## References

[1] Joshi PA, Di Grappa MA, Khokha R (2012). Active allies: hormones, stem cells and the niche in adult mammopoiesis. Trends Endocrinol. Metab..

[2] Stingl J, Caldas C (2007). Molecular heterogeneity of breast carcinomas and the cancer stem cell hypothesis. Nat. Rev. Cancer.

[3] Stingl J (2006). Purification and unique properties of mammary epithelial stem cells. Nature.

[4] Shackleton M (2006). Generation of a functional mammary gland from a single stem cell. Nature.

[5] Van Keymeulen A (2011). Distinct stem cells contribute to mammary gland development and maintenance. Nature.

[6] Rios AC, Fu NY, Lindeman GJ, Visvader JE (2014). In situ identification of bipotent stem cells in the mammary gland. Nature.

[7] Wang D (2015). Identification of multipotent mammary stem cells by protein C receptor expression. Nature.

[8] Wang C, Christin JR, Oktay MH, Guo W (2017). Lineage-biased stem cells maintain estrogen-receptor-positive and -negative mouse mammary luminal lineages. Cell Rep..

[9] van Amerongen R, Bowman AN, Nusse R (2012). Developmental stage and time dictate the fate of Wnt/beta-catenin-responsive stem cells in the mammary gland. Cell. Stem. Cell..

[10] Joshi PA (2010). Progesterone induces adult mammary stem cell expansion. Nature.

[11] Joshi PA (2015). RANK signaling amplifies WNT-responsive mammary progenitors through R-SPONDIN1. Stem Cell Rep..

[12] Casey AE (2018). Mammary molecular portraits reveal lineage-specific features and progenitor cell vulnerabilities. J. Cell. Biol..

[13] Parmar H, Cunha GR (2004). Epithelial-stromal interactions in the mouse and human mammary gland in vivo. Endocr. Relat. Cancer.

[14] Wiseman BS, Werb Z (2002). Stromal effects on mammary gland development and breast cancer. Science.

[15] Houthuijzen JM, Jonkers J (2018). Cancer-associated fibroblasts as key regulators of the breast cancer tumor microenvironment. Cancer Metastas-. Rev..

[16] Barcellos-Hoff MH, Ravani SA (2000). Irradiated mammary gland stroma promotes the expression of tumorigenic potential by unirradiated epithelial cells. Cancer Res..

[17] Maffini MV, Soto AM, Calabro JM, Ucci AA, Sonnenschein C (2004). The stroma as a crucial target in rat mammary gland carcinogenesis. J. Cell. Sci..

[18] Gyorki DE, Asselin-Labat ML, van Rooijen N, Lindeman GJ, Visvader JE (2009). Resident macrophages influence stem cell activity in the mammary gland. Breast Cancer Res..

[19] Zhao C (2017). Stromal Gli2 activity coordinates a niche signaling program for mammary epithelial stem cells. Science.

[20] Landskroner-Eiger S, Park J, Israel D, Pollard JW, Scherer PE (2010). Morphogenesis of the developing mammary gland: stage-dependent impact of adipocytes. Dev. Biol..

[21] Couldrey C (2002). Adipose tissue: a vital in vivo role in mammary gland development but not differentiation. Dev. Dyn..

[22] Hepler C, Vishvanath L, Gupta RK (2017). Sorting out adipocyte precursors and their role in physiology and disease. Genes Dev..

[23] Rodeheffer MS, Birsoy K, Friedman JM (2008). Identification of white adipocyte progenitor cells in vivo. Cell.

[24] Lee YH, Petkova AP, Mottillo EP, Granneman JG (2012). In vivo identification of bipotential adipocyte progenitors recruited by beta3-adrenoceptor activation and high-fat feeding. Cell. Metab..

[25] Berry R, Rodeheffer MS (2013). Characterization of the adipocyte cellular lineage in vivo. Nat. Cell Biol..

[26] Andrae J, Gallini R, Betsholtz C (2008). Role of platelet-derived growth factors in physiology and medicine. Genes Dev..

[27] Hoch RV, Soriano P (2003). Roles of PDGF in animal development. Development.

[28] Festa E (2011). Adipocyte lineage cells contribute to the skin stem cell niche to drive hair cycling. Cell.

[29] Macias H, Hinck L (2012). Mammary gland development. Wiley Interdiscip. Rev. Dev. Biol..

[30] Shehata M (2012). Phenotypic and functional characterization of the luminal cell hierarchy of the mammary gland. Breast Cancer Res..

[31] Sleeman KE (2007). Dissociation of estrogen receptor expression and in vivo stem cell activity in the mammary gland. J. Cell. Biol..

[32] Asselin-Labat ML (2010). Control of mammary stem cell function by steroid hormone signalling. Nature.

[33] Visvader JE, Stingl J (2014). Mammary stem cells and the differentiation hierarchy: current status and perspectives. Genes Dev..

[34] Hudak CS (2014). Pref-1 marks very early mesenchymal precursors required for adipose tissue development and expansion. Cell Rep..

[35] Gulyaeva O, Nguyen H, Sambeat A, Heydari K, Sul HS (2018). Sox9-Meis1 inactivation is required for adipogenesis, advancing Pref-1(+) to PDGFRalpha(+) cells. Cell Rep..

[36] Jeffery E (2014). Characterization of Cre recombinase models for the study of adipose tissue. Adipocyte.

[37] Joshi PA, Chang H, Hamel PA (2006). Loss of Alx4, a stromally-restricted homeodomain protein, impairs mammary epithelial morphogenesis. Dev. Biol..

[38] De Pascalis C, Etienne-Manneville S (2017). Single and collective cell migration: the mechanics of adhesions. Mol. Biol. Cell.

[39] Maritzen T, Schachtner H, Legler DF (2015). On the move: endocytic trafficking in cell migration. Cell. Mol. Life Sci..

[40] Bunney TD, Katan M (2010). Phosphoinositide signalling in cancer: beyond PI3K and PTEN. Nat. Rev. Cancer.

[41] Fraser JK, Wulur I, Alfonso Z, Hedrick MH (2006). Fat tissue: an underappreciated source of stem cells for biotechnology. Trends Biotechnol..

[42] Safford KM (2002). Neurogenic differentiation of murine and human adipose-derived stromal cells. Biochem. Biophys. Res. Commun..

[43] Lue J (2010). Transdifferentiation of adipose-derived stem cells into hepatocytes: a new approach. Liver Int..

[44] Morroni M (2004). Reversible transdifferentiation of secretory epithelial cells into adipocytes in the mammary gland. Proc. NatlAcad. Sci. USA.

[45] Ishay-Ronen D (2019). Gain fat-lose metastasis: converting invasive breast cancer cells into adipocytes inhibits cancer metastasis. Cancer Cell..

[46] Vona-Davis L, Howard-McNatt M, Rose DP (2007). Adiposity, type 2 diabetes and the metabolic syndrome in breast cancer. Obes. Rev..

[47] Renehan AG, Zwahlen M, Egger M (2015). Adiposity and cancer risk: new mechanistic insights from epidemiology. Nat. Rev. Cancer.

[48] Zeve D, Tang W, Graff J (2009). Fighting fat with fat: the expanding field of adipose stem cells. Cell. Stem. Cell..

[49] Park J, Morley TS, Kim M, Clegg DJ, Scherer PE (2014). Obesity and cancer--mechanisms underlying tumour progression and recurrence. Nat. Rev. Endocrinol..

[50] Bertolini F, Orecchioni S, Petit JY, Kolonin MG (2014). Obesity, proinflammatory mediators, adipose tissue progenitors, and breast cancer. Curr. Opin. Oncol..

[51] Morsing M (2016). Evidence of two distinct functionally specialized fibroblast lineages in breast stroma. Breast Cancer Res..

[52] Cardiff RD, Wellings SR (1999). The comparative pathology of human and mouse mammary glands. J. Mammary Gland Biol. Neoplasia.

[53] Nolan E (2016). RANK ligand as a potential target for breast cancer prevention in BRCA1-mutation carriers. Nat. Med..

[54] Sigl V (2016). RANKL/RANK control Brca1 mutation-driven mammary tumors. Cell Res..

[55] Kelsey JL, Gammon MD, John EM (1993). Reproductive factors and breast cancer. Epidemiol. Rev..

[56] van Kruijsdijk RC, van der Wall E, Visseren FL (2009). Obesity and cancer: the role of dysfunctional adipose tissue. Cancer Epidemiol. Biomark. Prev..

[57] Dobin A (2013). STAR: ultrafast universal RNA-seq aligner. Bioinformatics.

[58] Ritchie ME (2015). limma powers differential expression analyses for RNA-sequencing and microarray studies. Nucleic Acids Res..

[59] Reimand J (2016). g:Profiler-a web server for functional interpretation of gene lists (2016 update). Nucleic Acids Res..

