# Supplementary material for: PDGFRα+ stromal adipocyte progenitors transition into epithelial cells during lobulo-alveologenesis in the murine mammary gland
Source: Nat Commun. 2019 Apr 15;10:1760. doi: 10.1038/s41467-019-09748-z (PMC6465250; doi:10.1038/s41467-019-09748-z)
Supplement: Supplementary file 1 — Supplementary Information [file 41467_2019_9748_MOESM1_ESM.pdf]

PDGFRα^+ mesenchymal adipocyte progenitors are a source of epithelial cells in the murine mammary gland

Joshi et al.

Supplementary Information

(Supplementary Figures 1-8 and Table 1)

Joshi et al Supplementary Figure 1

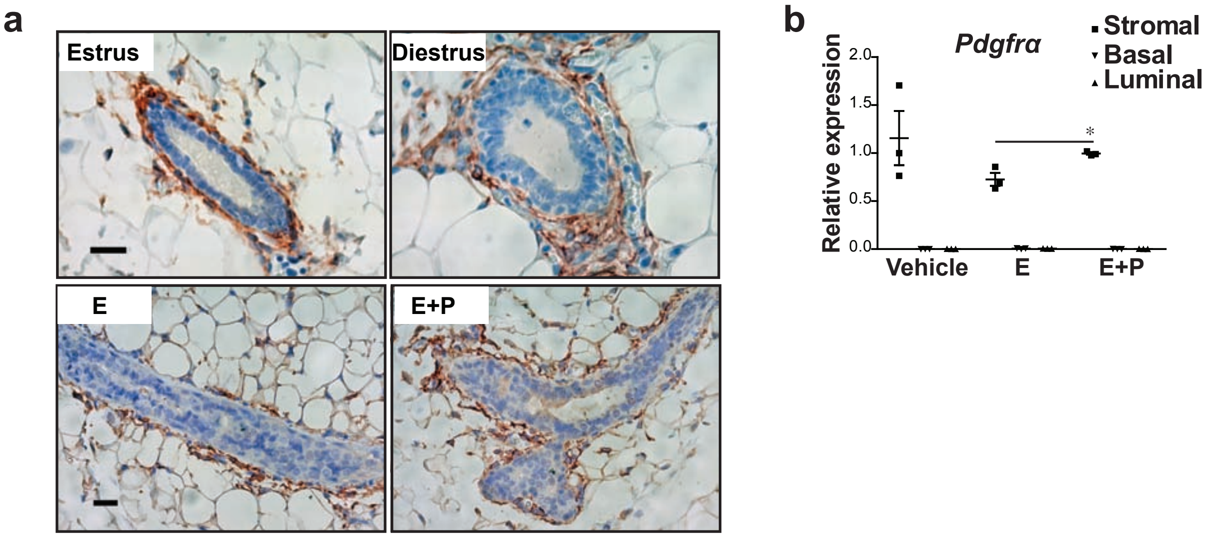

Pattern of PDGFRα expression in the adult mouse mammary gland. (a) Immunohistochemistry for PDGFRα during the estrous cycle (top panels) and hormone treatment with estrogen (E) or estrogen+progesterone (E+P) (bottom panels); scale bar = 20 μm. **(b)** Stromal ($CD49f^{-}EpCAM^{-}$) and luminal ($CD49f^{lo}EpCAM+$), basal ($CD49f^{hi}EpCAM^{+}$) mammary epithelial cells were FACS-purified from ovariectomized mice treated with vehicle, estrogen (E) or estrogen+progesterone (E+P) and analyzed by qRT-PCR for Pdgfrα relative to β-actin (n = 3 mice per group). Data represent mean ± s.e.m. *$p < 0.05$ (one-way ANOVA). Source data are provided as a Source Data file.

Joshi et al Supplementary Figure 2

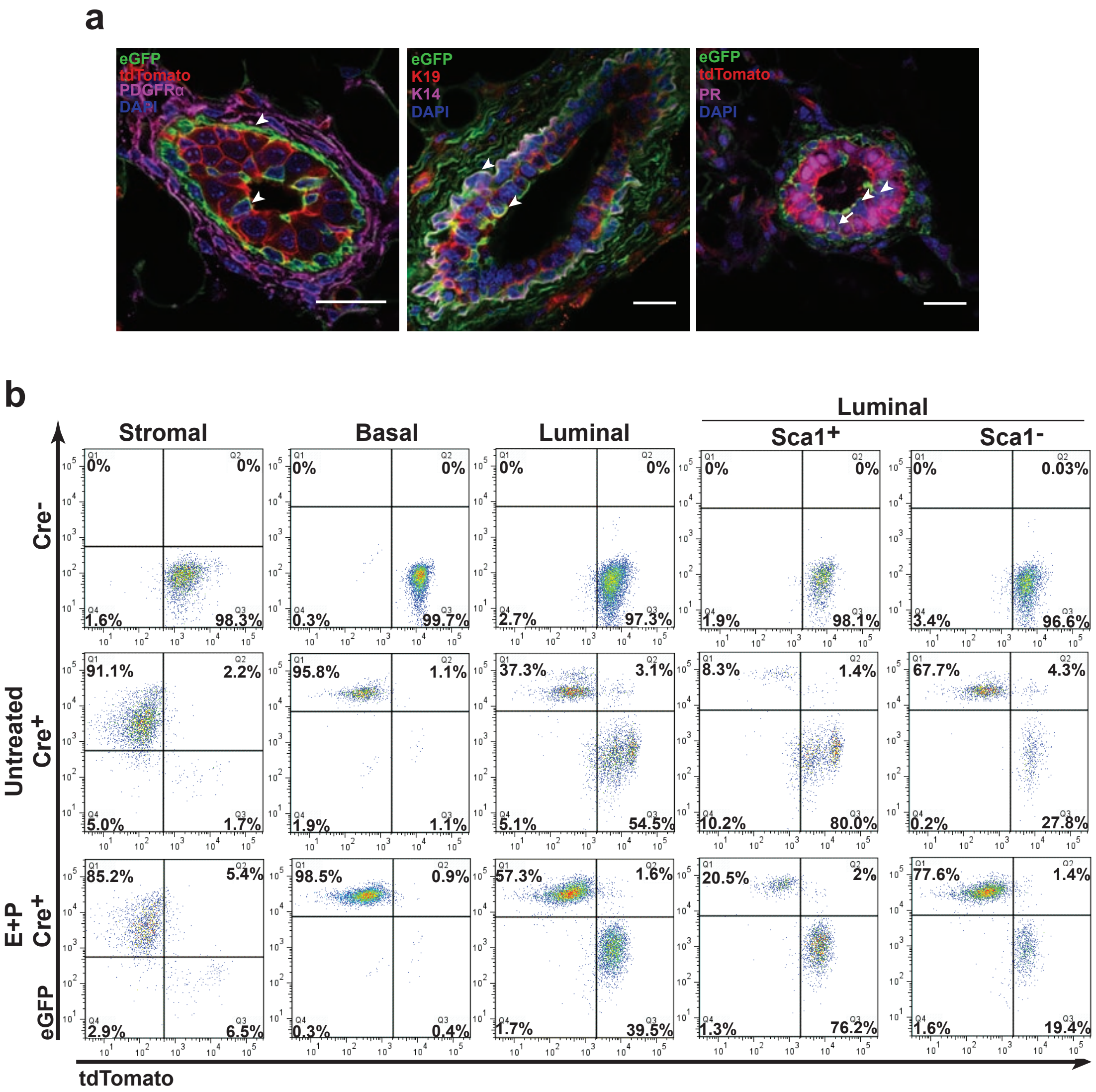

Adult *PdgfraCre R26mTmG* mammary epithelium exhibits GFP+ cells. (a) GFP+ cells with PDGFRα (left), basal (K14) and luminal (K19)-specific epithelial markers (middle) and progesterone receptor (PR; right) in mammary tissue sections from untreated mice (representative of n = 3 mice and 5 fields per tissue section); scale bar = 25 µm. White arrowheads indicate GFP+ cells that are either K14+, K19+ or PR- and arrow indicates a GFP+ PR+ cell. **(b)** Representative FACS plots of GFP+ and tdTomato+ cells in mammary subsets of adult glands isolated from untreated (n = 3) or E+P treated (n = 2) mice.

Joshi et al Supplementary Figure 3

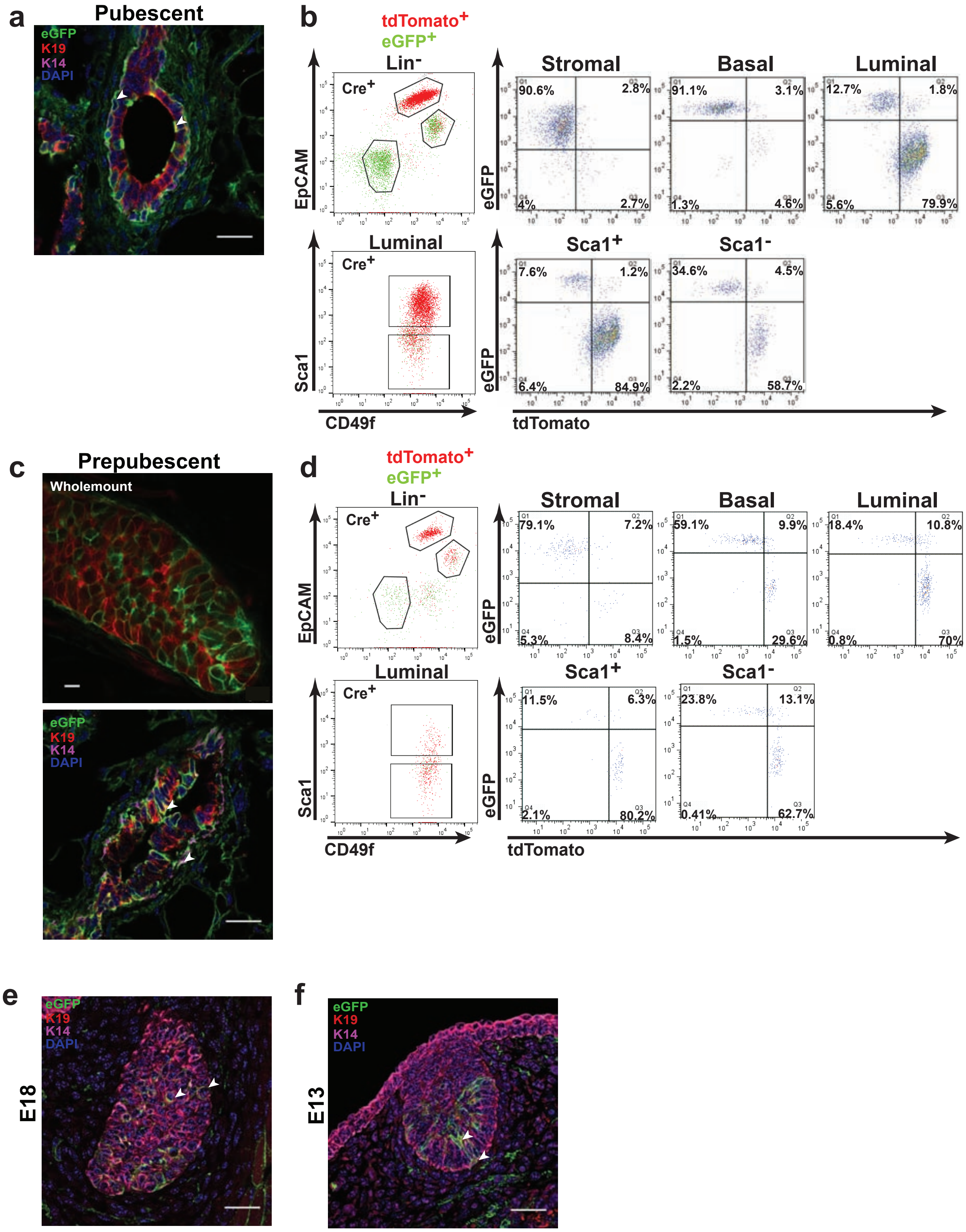

GFP progeny from Pdgfrα$^+$ cell tracing are found within mammary epithelial subsets during early development. **(a)** Immunostained image of GFP$^+$ cells with luminal (K19)- and basal (K14)-specific epithelial markers in a pubescent 5 week old gland (representative of n = 3 mice and 5 fields per tissue section); scale bar = 25 µm. **(b)** Representative FACS plots of GFP$^+$ and tdTomato$^+$ cells in mammary subsets from pubescent mice (n = 3 mice). **(c)** Native GFP and tdTomato fluorescence of a prepubescent ductal tip in a wholemount (top) and immunostained image of GFP$^+$ cells with epithelial markers in prepubescent 2 week old mammary tissue (bottom); scale bar = 10µm (whole mount) and 25 µm (tissue section); representative of n = 3 individual mice. **(d)** Representative FACS plots of mammary subsets from prepubescent mice (n = 3). GFP$^+$ cells with epithelial keratins in the primitive gland at embryonic day 18 **(e)** or day 13 **(f)** (n = 3 mice per stage); scale bar = 25 µm. White arrowheads indicate GFP$^+$ cells that are K19$^+$ or K14$^+$.

Joshi et al Supplementary Figure 4

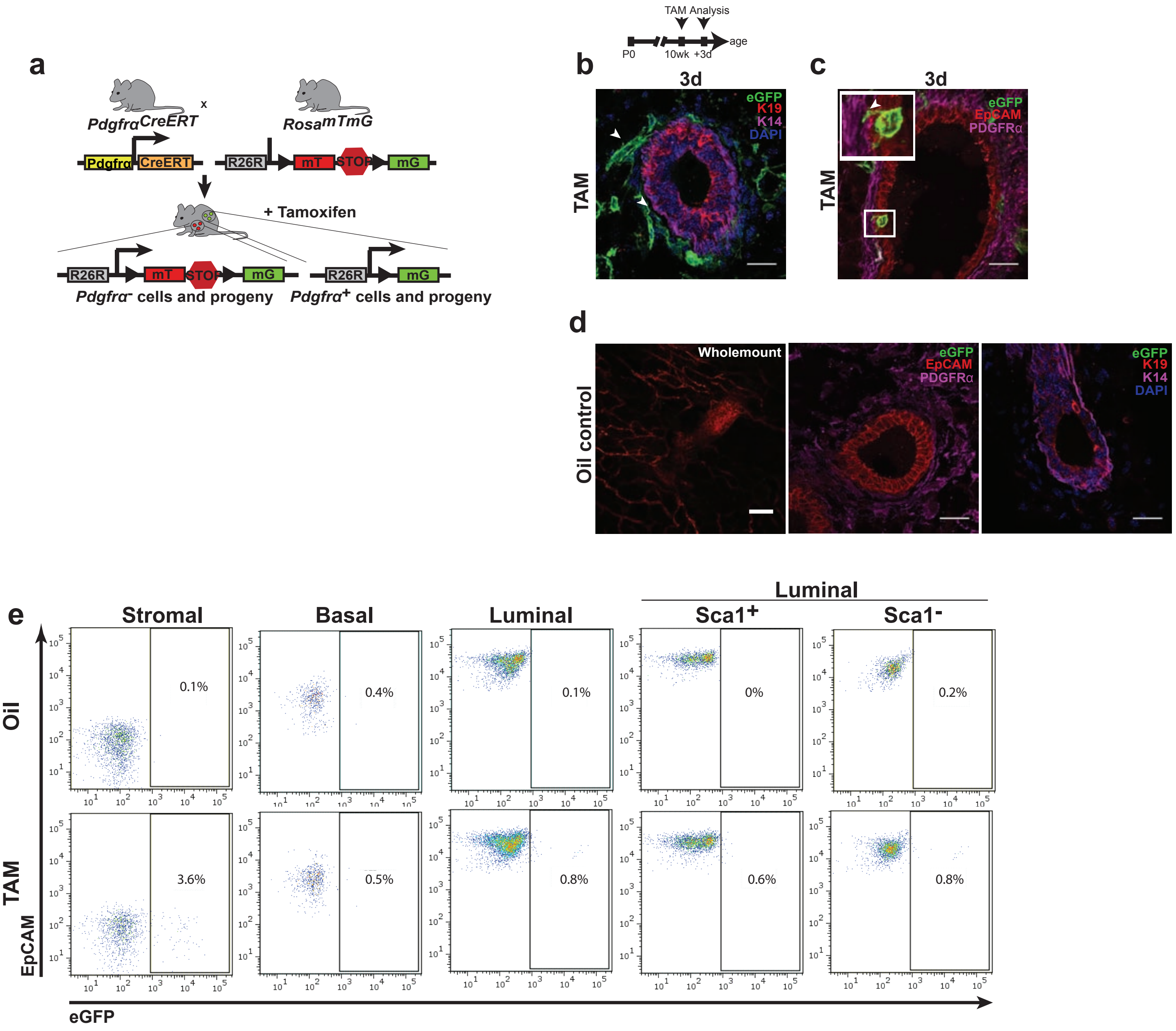

Inducible tracing of Pdgfrα⁺ cells in the adult mammary gland. (a) A tamoxifen-inducible lineage tracing model driven by the Pdgfrα promoter. **(b)** Mammary tissue section after a 3d short trace following Tamoxifen (TAM) induction and immunostained for GFP with epithelial keratins showing stromal restriction of GFP⁺ cells (representative of n = 3 mice and 5 fields per tissue section). **(c)** Rare GFP⁺ cells that have entered the epithelial space but still in contact with PDGFRα⁺ stroma; scale bar = 25 μm for (b,c). **(d)** Wholemount (scale bar = 50 μm) and immunofluoresent images (middle and right; scale bar = 25 μm) of GFP, PDGFRα and epithelial markers (EpCAM, K19-Luminal, K14-basal) in tissue sections from oil injected controls. **(e)** FACS plots of GFP-labelled cells within mammary subsets after the 3d trace (representative of n = 3 mice).

Joshi et al Supplementary Figure 5

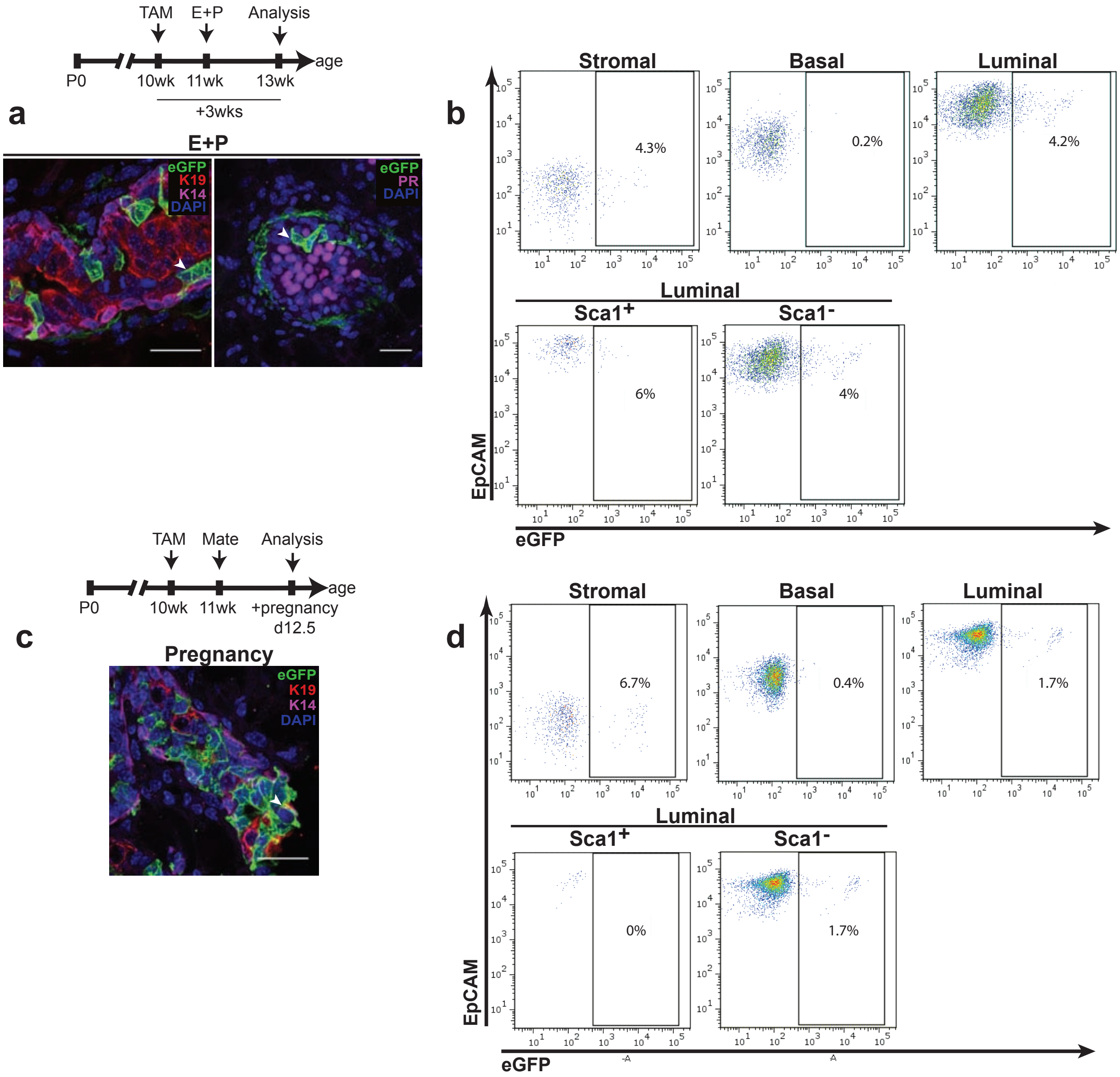

GFP-labelled cells following Cre induction in adult *PdgfraCreERT R26mTmG* glands participate in epithelial expansion driven by hormones and pregnancy. (a) Immunofluorescent images of GFP with epithelial Keratins (left) and Progesterone receptor (PR; right) after a three week trace following TAM induction and hormone stimulation (representative of n = 3 mice and 5 fields per tissue section); scale bar = 25 μm. **(b)** Representative FACS plots of GFP^+ cells within mammary subpopulations after hormone stimulation. **(c)** Image of GFP^+ cells with epithelial Keratins in adult mammary tisssue during pregnancy (representative of n = 3 mice and 5 fields per tissue section); scale bar = 25 μm. **(d)** FACS plots of GFP^+ cells in mammary subpopulations after cell tracing during pregnancy.

Joshi et al Supplementary Figure 6

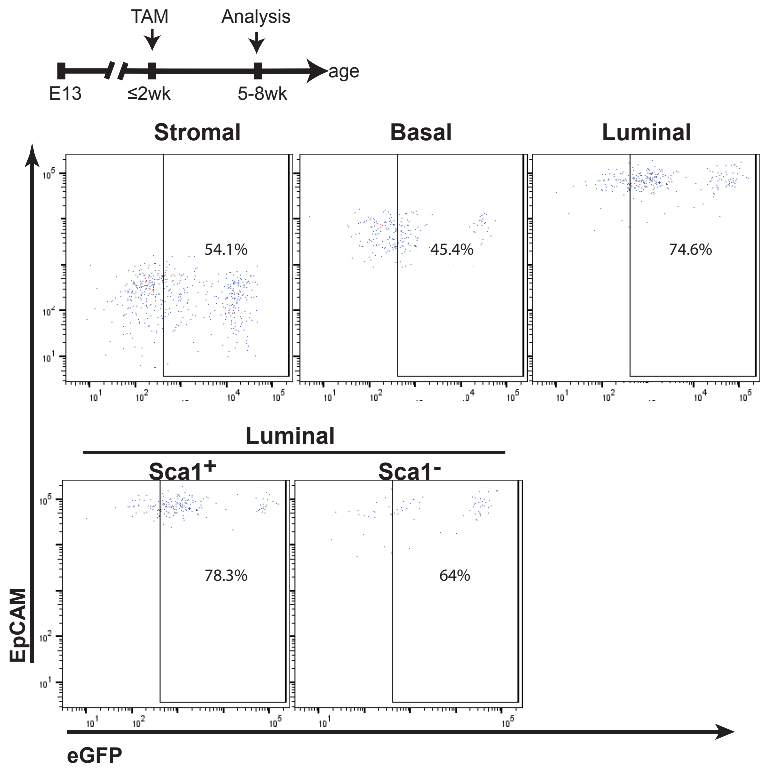

GFP+ cells following Cre induction in prepubescent *PdgfrαCreERT R26mTmG* mice.
FACS plots of GFP$^+$ cells in different mammary subsets following early labelling (n = 3 individual mice).

Joshi et al Supplementary Figure 7

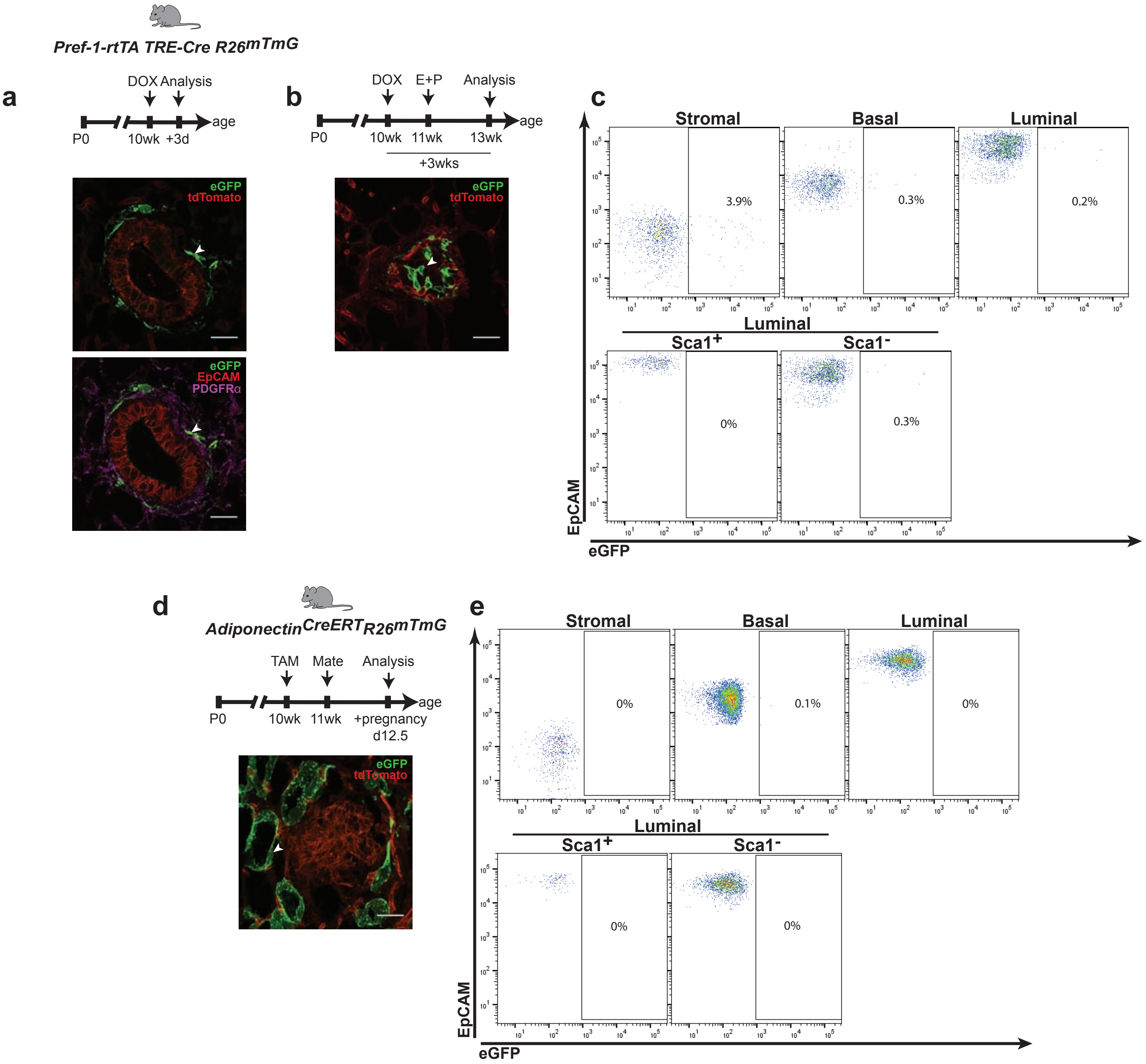

Adipocyte progenitors but not mature adipocytes are recruited into the adult mammary epithelium.
(a) Immunofluorescent images of GFP-labelled cells with tdTomato (top), EpCAM and PDGFRα (bottom) in mammary tissue sections derived from Pref-1 lineage tracing mice 3 days following doxycycline (DOX) induction (representative of n = 2 mice and 5 fields per tissue section). **(b)** GFP-labelled cells in Pref-1 mice are found in the mammary epithelium following a 3 week trace involving hormone stimulation (representative of n = 3 mice and 5 fields per tissue section). **(c)** Representative FACS plots of GFP^{+} cell distribution in mammary subpopulations after hormone stimulation (n = 3 mice). **(d,e)** Immunostaining and flow cytometry analysis show that GFP-labelled cells in Adiponectin lineage tracing mice remain restricted to adipocytes during pregnancy (representative of n = 3 mice). Arrowheads indicate GFP^{+} cells; Scale bar = 25 μm in (a,b,d).

Joshi et al Supplementary Figure 8

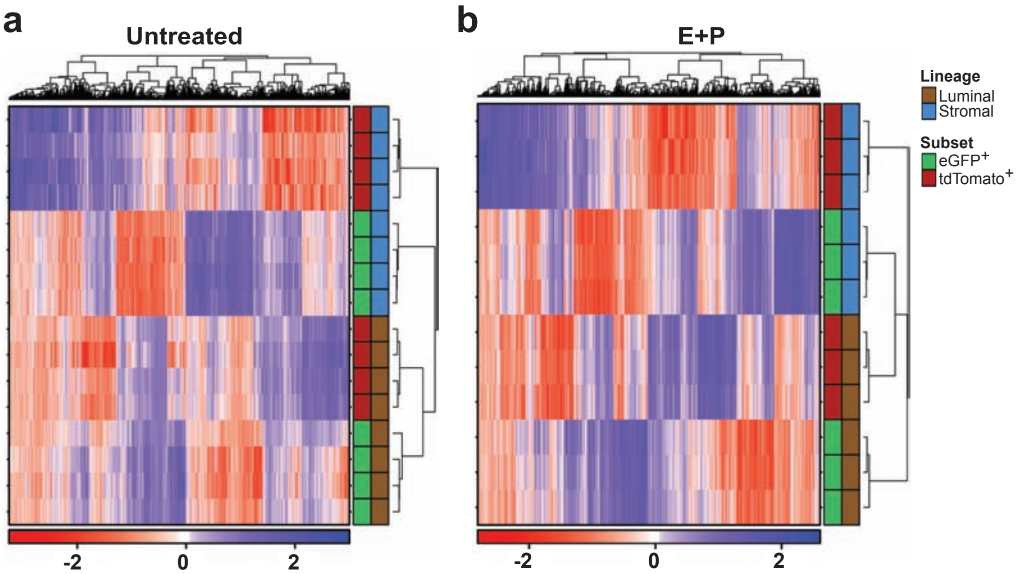

RNA-seq analysis of eGFP⁺ vs. tdTomato⁺ cells from *PdgfraCre R26mTmG* mammary glands. **(a,b)** Heat maps showing clustering of differentially expressed genes between eGFP⁺ and tdTomato⁺ cell populations in stromal and luminal compartments from untreated and E+P-stimulated mice (n = 4 untreated and n = 3 E+P treated mice). Source data are available as a Source Data file.

Joshi et al Supplementary Table 1

Primers and probes for droplet digital PCR

Oligo	Sequence	Fluor/Quencher
*Gapdh*F	AGGTTGTCTCCTGCGACT	
*Gapdh*R	TGCTGTAGCCGTATTCATTGTCA	
*Gapdh*P	ACTCCCACTCTTCCACCTTCGATGC	FAM/ZEN/BHQ1
*Pdgfra*F	TGCAGTTGCCTTACGACTCCA	
*Pdgfra*R	CCATAAGCTGTACCTTCGACC	
*Pdgfra*P	CGACCAAGCACGAGGCCATCTCT	HEX/ZEN/BHQ2
*K18*F	CCGCCTTGCCGCCGATG	
*K18*R	CTACCACCTTGCGGAGTCCAT	
*K18*P	CGCTCTCCACAGACTGGCGCAT	HEX/ZEN/BHQ2
*K14*F	ATTCTCCTCTGGCTCTCAGTC	
*K14*R	TGGAGACCACCTTGCCAT	
*K14*P	CCTCCACCAACCGCCAGATCCGC	FAM/ZEN/BHQ1
