# Supplementary material for: PDGFRα+ stromal adipocyte progenitors transition into epithelial cells during lobulo-alveologenesis in the murine mammary gland
Source: Nat Commun. 2019 Apr 15;10:1760. doi: 10.1038/s41467-019-09748-z (PMC6465250; doi:10.1038/s41467-019-09748-z)
Supplement: Supplementary file 2 — Description of Additional Supplementary Files [file 41467_2019_9748_MOESM2_ESM.docx]

**Title:** Supplementary Movie 1
**Description:** Optical sections through an adult Pdgfr­CreR26mTmG mammary wholemount presented in Fig. 2b.

**Title:**  Supplementary Movie 2
**Description:** Optical sections through an adult TAM‐induced, hormone‐stimulated Pdgfr­CreERTR26mTmGmammary wholemount presented in Fig. 4e.

**Title:** Supplementary Movie 3
**Description:** 3D rendering of a Z stack acquired from imaging an immunostained adult Pdgfr­CreERTR26mTmG mammary tissue section presented in Fig. 4e. Tissue was stained for GFP (green), tdTomato (red) and PDGFR­­(magenta).

**Title:** Supplementary Movie 4
**Description:** 3D visualization of a transplanted wild‐type non‐reporter mammary epithelial outgrowth arising in a Pdgfr­CreERTR26mTmG cleared fat pad that was TAM‐induced and hormone treated after transplantation, presented in Fig.6b. Tissue was stained for GFP (green), EpCAM (red) and DAPI (blue).

**Title:** Supplementary Movie 5
**Description:** Time lapse imaging of GFP+ fibroblasts isolated from Pdgfr­CreR26mTmG mammary glands co‐cultured with tdTomato+ epithelial organoids from a Cre‐ R26mTmGmammary gland and treated with PDGFCC as shown in Fig. 7h.

**Title:** Supplementary Movie 6
**Description:** Time lapse imaging of GFP+ fibroblasts isolated from Pdgfr­CreR26mTmG mammary glands co‐cultured with tdTomato+ epithelial organoids from a Cre‐ R26mTmGmammary gland and treated with vehicle control a shown in Fig. 7h
