# Supplementary material for: PDGFRα+ stromal adipocyte progenitors transition into epithelial cells during lobulo-alveologenesis in the murine mammary gland
Source: Nat Commun. 2019 Apr 15;10:1760. doi: 10.1038/s41467-019-09748-z (PMC6465250; doi:10.1038/s41467-019-09748-z)
Supplement: Supplementary file 9 — Peer Review File [file 41467_2019_9748_MOESM9_ESM.pdf]

Editorial Note: This manuscript has been previously reviewed at another journal that is not operating a transparent peer review scheme. The manuscript was considered suitable for publication without further review at Nature Communications.
